# The Post-COVID syndrome caused by excessive inflammation: pathogenesis, potential targets and therapeutic agents

**DOI:** 10.3389/fphar.2026.1852220

**Published:** 2026-08-05

**Authors:** Tao Zhou, Guotao Xue, Yifan Zhang, Mengqi Li, Dingkun Zhang, Junzhi Lin, Chuanhong Luo, Li Han, Jinna Tian, Haozhou Huang

**Affiliations:** 1 Chinese Medicine Germplasm Resources Innovation and Effective Uses Key Laboratory of Sichuan Province, School of Pharmacy, Chengdu University of Traditional Chinese Medicine, Chengdu, China; 2 School of Modern Chinese Medicine Industry, Chengdu University of Traditional Chinese Medicine, Chengdu, China; 3 Sichuan Nursing Vocational College, Chengdu, China; 4 Sichuan Provincial Engineering Research Center of Innovative Re-Development of Famous Classical Formulas, Tianfu TCM Innovation Harbour, Chengdu University of Traditional Chinese Medicine, Chengdu, China; 5 TCM Regulating Metabolic Diseases Key Laboratory of Sichuan Province, Hospital of Chengdu University of Traditional Chinese Medicine, Chengdu, China; 6 Affiliated Hospital of Chengdu University of Traditional Chinese Medicine, Chengdu, China

**Keywords:** chronic fatigue, excessive inflammation, pathogenesis, post-COVID syndrome, therapeutic agents, therapeutic targets

## Abstract

Post-COVID syndrome is predominantly characterized by persistent fatigue that cannot be attributed to other underlying diseases or relieved by conventional rest. Such symptoms may last for weeks to months, substantially impairing patients’ health status and quality of life. Nevertheless, current understanding of the pathogenesis of post-COVID syndrome remains insufficient, posing major challenges to its effective clinical management. Therefore, this review elaborates comprehensively on the pathogenesis, potential therapeutic targets, and pharmacological interventions for post-COVID syndrome, aiming to provide evidence for drug research and development as well as clinical application. Four interrelated pathogenic mechanisms sustaining chronic inflammation after viral clearance are collectively involved in disease progression, including immune dysregulation accompanied by chronic low-grade inflammation, mitochondrial dysfunction and impaired energy metabolism, renin-angiotensin system imbalance, and gut microbiota dysbiosis with disrupted gut-brain axis function. Based on these mechanistic findings, this review highlights potential therapeutic targets such as TLR4/TLR7, the JAK/STAT pathway, the NLRP3 inflammasome, the AMPK/NRF2 axis, and the PINK1/Parkin pathway. Accordingly, we summarize mechanism-targeted candidate drugs, including Toll-like receptor antagonists, JAK inhibitors, probiotics, mitochondria-targeted agents and drugs correcting renin-angiotensin system imbalance. In addition, this review summarizes other therapeutic candidates acting via distinct mechanisms, such as fluvoxamine, coenzyme Q10 combined with alpha-lipoic acid, astragalus root extracts, other medicinal herbs and traditional Chinese compound formulas. Post-COVID syndrome represents a complex public health challenge. Further in-depth mechanistic research and rational application of emerging technologies are still required to develop therapeutic strategies for preventing and alleviating the onset and progression of post-COVID syndrome.

## 1 Introduction

Although significant progress has been made in the understanding and clinical management of COVID-19, its long-term health sequelae remain a public health challenge that demands urgent attention (Visconti et al., 2025). Long COVID, also known as post-COVID-19 condition or post-acute sequelae of SARS-CoV-2 infection, is a prevalent health disorder. According to the WHO, Long COVID refers to symptoms that emerge 3 months after SARS-CoV-2 infection, last for at least 2 months and cannot be explained by alternative medical conditions. The most common symptoms include fatigue, dyspnea, and sleep disturbances. It presents a wide spectrum of clinical manifestations, among which persistent fatigue is the most prominent and prevalent symptom. Accordingly, this review focuses on exploring post-COVID syndrome (PCS) predominantly characterized by persistent fatigue.

**FIGURE 1:**
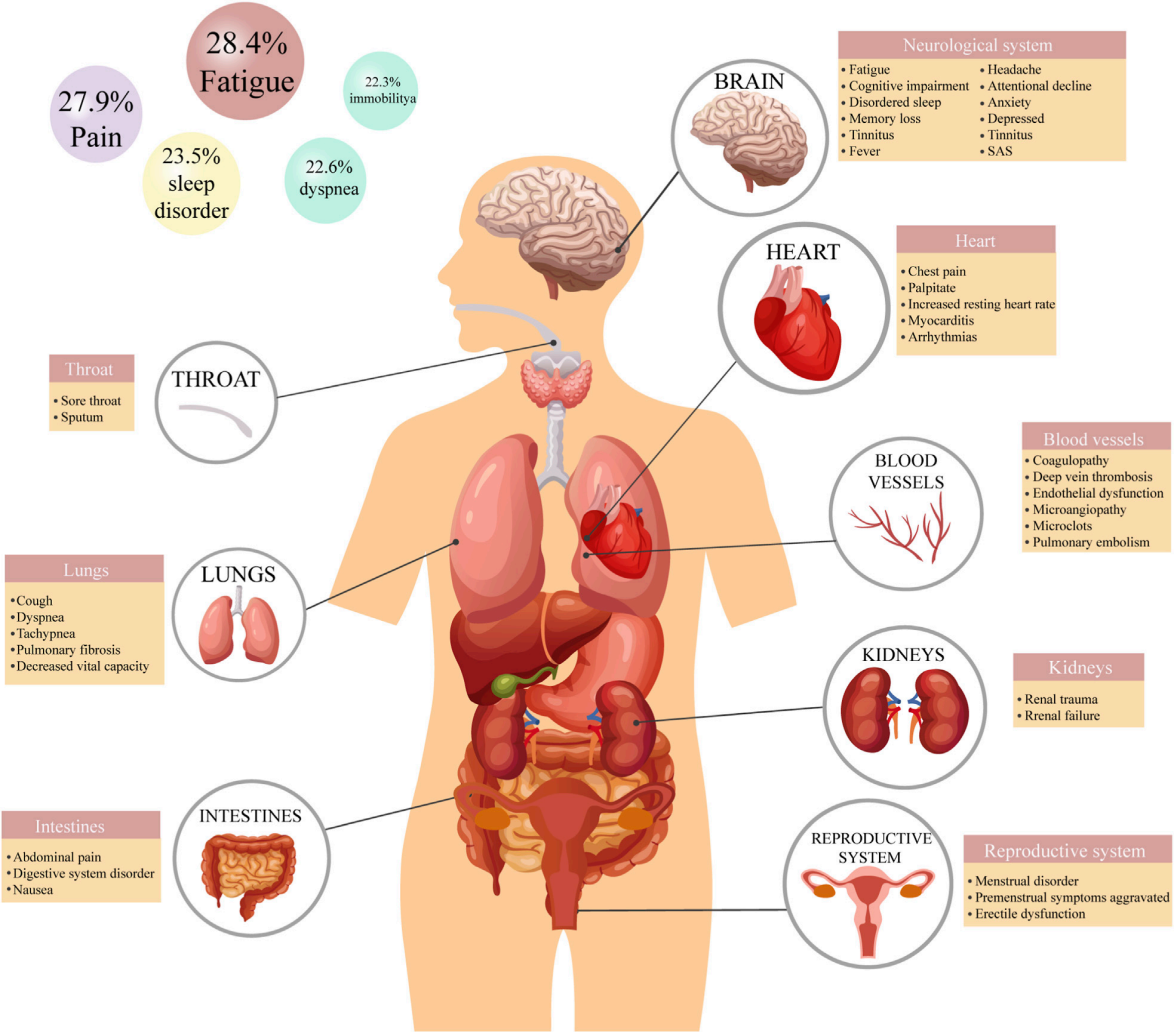
Long-term effects of the post-COVID syndrome. Symptoms that may affect the nervous, cardiovascular, renal and reproductive systems, demonstrating the incidence of common systemic symptoms such as fatigue and pain.

Large-scale epidemiological studies have reported a high prevalence of PCS. A study co-conducted by the WHO and the University of Washington found that 3 months after symptomatic SARS-CoV-2 infection, 6.2% of individuals experienced at least one sequela, of which 3.7% had persistent respiratory problems, 3.2% had persistent fatigue accompanied by physical pain or mood swings, 2.2% developed cognitive impairment, and 0.9% still had sequelae after 1 year (Hanson et al., 2022). A systematic review and meta-analysis covering 194 studies and 735,006 participants showed that at least 45% of people infected with SARS-CoV-2 still had one or more symptoms during a 4-month follow-up period with a mean follow-up of 126 days, regardless of hospitalization status. The five most common symptoms were fatigue (28.4%), pain or discomfort (27.9%), sleep disturbance (23.5%), dyspnea (22.6%), and activity limitation (22.3%). Even among non-hospitalized individuals, fatigue remains one of the most prevalent symptoms of COVID-19, with a prevalence rate as high as 34.8% (O’Mahoney et al., 2023). Other long-term effects of PCS are presented in Figure 1. In addition, a survey of 74,075 Chinese residents over 1 year, with 68,200 valid responses, revealed that the most frequent long COVID symptoms included fatigue (30.53%), memory decline (27.93%), reduced exercise capacity (18.29%), and brain fog (16.87%) (Qin et al., 2024). Persistent fatigue greatly limits patients’ daily activities and work efficiency. Long-term fatigue is accompanied by a higher incidence of sleep disorders, depression, and anxiety, which further impairs mental health and exacerbates the condition. Given the comprehensive harm caused by PCS, research on its pathogenesis and therapeutic interventions is particularly urgent and necessary.

The current understanding of the pathological mechanisms of PCS is gradually deepening. Unlike classical pro-inflammatory immune activation, accumulating evidence suggests that a core driver may be an aberrant anti-inflammatory state secondary to excessive inflammatory responses, namely, a disease pattern dominated by alternatively activated M2 macrophages (Kovarik et al., 2023). During the acute phase of SARS-CoV-2 infection, a strong inflammatory storm can trigger a compensatory anti-inflammatory response. However, when this anti-inflammatory response becomes excessively sustained, M2 macrophages secrete large amounts of anti-inflammatory cytokines such as IL-10 and TGF-β, creating an immunosuppressive and pro-fibrotic microenvironment. This leads to aberrant tissue repair and persistent low-grade inflammation, ultimately manifesting as sustained fatigue.

Our previous research on exercise-induced fatigue has confirmed that gut microbiota modulation, mitochondrial protection, and improved energy metabolism serve as crucial anti-fatigue mechanisms. While exercise-induced fatigue and PCS arise from distinct triggers, they exhibit striking pathological overlaps, such as mitochondrial dysfunction, disrupted energy metabolism, and gut microecological dysbiosis (Zhang et al., 2023). Several high-quality recent reviews further demonstrate that mitochondrial dysfunction and microbiome dysregulation constitute core pathological mechanisms of PCS (Baalbaki et al., 2025). In addition, microbiome perturbations can alter neural function through the gut-brain axis, suggesting that these pathways merit extensive attention in PCS research. In fact, beyond the aforementioned pathways, immune dysregulation and renin-angiotensin system (RAS) imbalance also contribute to PCS pathogenesis (Hashimoto, 2023). On this basis, this review summarizes the core mechanisms of PCS, including immune dysregulation with chronic inflammation, mitochondrial dysfunction and impaired energy metabolism, RAS imbalance, as well as gut microbiota dysbiosis and microbial-gut-brain (MGB) axis disruption. These pathological processes are not isolated but interact with each other, becoming self-sustaining after viral clearance and forming a vicious cycle of excessive inflammation, tissue damage and persistent fatigue. Nevertheless, systematic knowledge of key molecular targets within the above mechanisms remains insufficient, and to date a validated PCS-specific therapeutic regimen is still lacking (Wang et al., 2023).

Given the complexity and multi-target pathogenic nature of PCS, this review identifies targets associated with TLR4/TLR7, JAK/STAT pathway, NLRP3 inflammasome, AMPK/NRF2 axis, PINK1/Parkin pathway, RAS axis and MGB axis based on the four core pathological mechanisms mentioned above. On this foundation, it further summarizes candidate drugs, including mechanism-targeted agents and pharmaceuticals functioning through other pathways. Collectively, this review elaborates the pathological characteristics, pathogenesis and potential therapeutic targets of inflammation-mediated PCS, and thoroughly discusses the research progress and future prospects of relevant therapeutic candidates. It aims to provide scientific insights for the drug development and clinical translation of PCS.

## 2 Pathogenesis of PCS

The core pathogenesis of PCS lies in the excessive and self-sustaining inflammatory state triggered by SARS-CoV-2 infection. This chronic inflammation is initiated and maintained by a cascade of interrelated pathological processes, including immune dysregulation, mitochondrial dysfunction, RAS imbalance, and gut microbiota dysbiosis, all of which persist even after viral clearance. Collectively, these mechanisms ultimately result in structural and functional damage to multiple organ systems, which explains why symptoms may still emerge or persist despite antiviral treatment.

### 2.1 SARS-CoV-2 infection as the inciting event

PCS is triggered by SARS-CoV-2 infection, and its pathogenesis can be divided into two stages (Channappanavar et al., 2016; Trougakos et al., 2021). In the first stage, viral replication directly causes virus-mediated tissue damage. The severity of such damage largely determines the second stage, which is characterized by the recruitment and activation of effector immune cells, thereby triggering local and systemic inflammatory responses. Importantly, this inflammatory state may persist even after viral clearance, acting as the initial trigger for the long-term symptoms of PCS.

**FIGURE 2:**
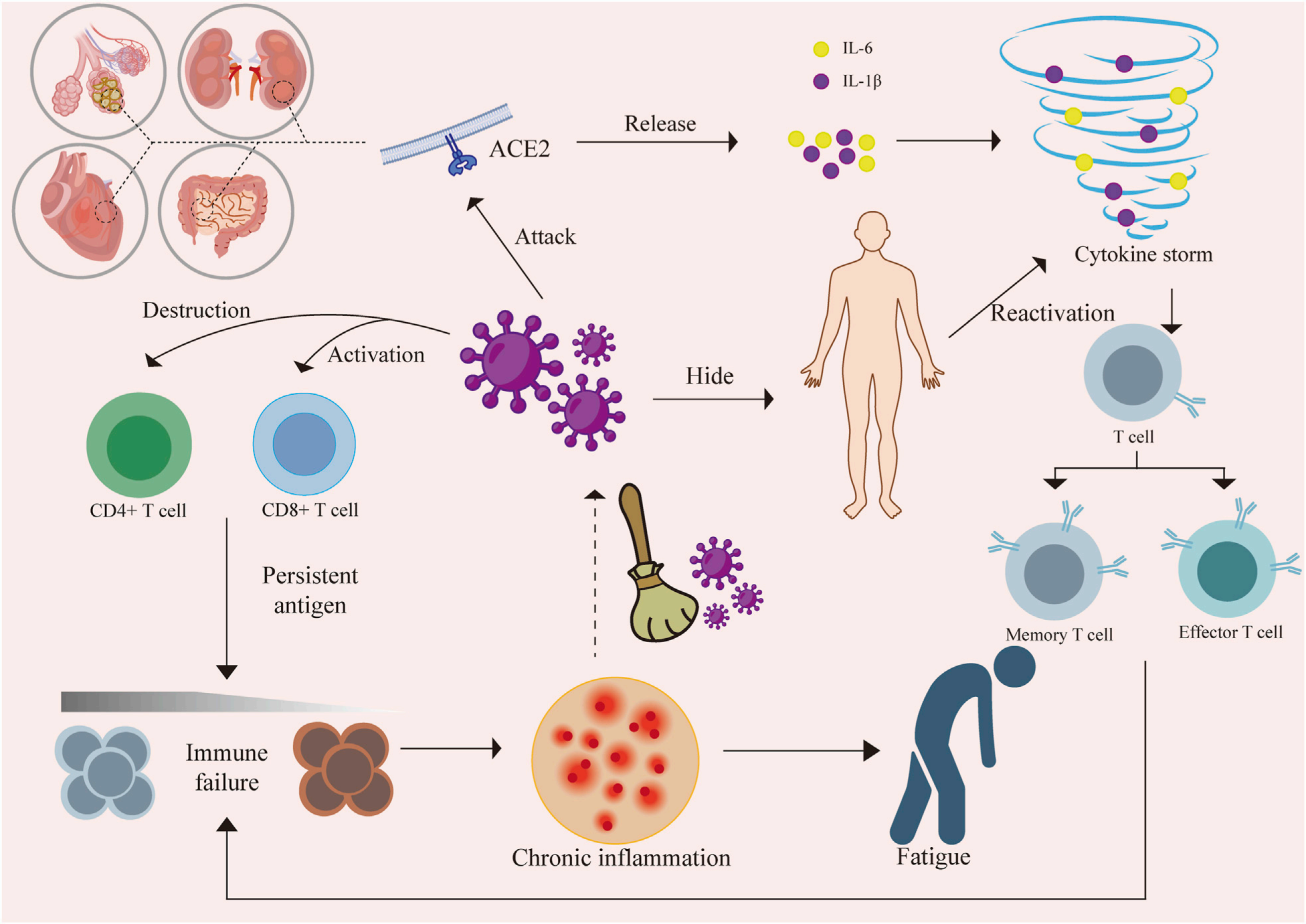
Viral infection mechanism of PCS. SARS-CoV-2 can attack ACE2 target cells, releasing viral and pro-inflammatory factors causing a cytokine storm that disrupts CD4^+^ T-cell function and promotes over-activation of CD8^+^ T-cells, leading to immune failure with inactivation of T-cell function, resulting in chronic inflammation and thus PCS.

SARS-CoV-2 invades host cells via the angiotensin-converting enzyme 2 (ACE2) receptor, which is expressed in alveolar epithelial cells, endothelial cells, cardiomyocytes, gastrointestinal epithelial cells, and renal cells. Infected cells release large amounts of viral particles and pro-inflammatory mediators, thereby inducing a cytokine storm and further activating the immune system (Ye et al., 2020). In some infected individuals, viral RNA or viral proteins can persist for months in tissues such as the gastrointestinal tract and lungs (Gareau and Barrett, 2023; Zuo et al., 2024), serving as a persistent source of antigenic stimulation to sustain pathological progression. The pathological processes induced by initial infection are summarized in Figure 2.

### 2.2 Immune dysregulation and chronic inflammation

The inflammatory microenvironment established following SARS-CoV-2 infection is further sustained and amplified through immune dysregulation. Viral infection can induce fever, which elevates systemic oxygen consumption and predisposes the body to relative hypoxia, thereby activating immune cells and upregulating HIF-1α expression. HIF-1α inhibits the activity of the pyruvate dehydrogenase complex, promoting the massive conversion of pyruvate to lactate catalyzed by lactate dehydrogenase, resulting in abnormal lactate accumulation and ultimately inducing generalized myalgia and fatigue. Meanwhile, infection further activates the pentose phosphate pathway to generate ribose and NADPH. NADPH acts on immune cells via NADPH oxidase and facilitates reactive oxygen species production, thereby exacerbating PCS-related clinical symptoms (Santos et al., 2021).

In addition to metabolic disorders, persistent antigenic stimulation triggered by residual viral components or persistent viral reservoirs can lead to immune exhaustion, characterized by dysfunction of antigen-specific T cells. SARS-CoV-2 impairs CD4^+^ T cell function and induces excessive activation of CD8^+^ T cells, thereby weakening antiviral immune defense, provoking sustained viral antigen stimulation, and ultimately driving T cell exhaustion (Mohammed et al., 2022). Exhausted T cells fail to clear residual virus but continuously secrete pro-inflammatory cytokines such as IL-6 and IL-1β (Simmonds et al., 2021), sustaining a tissue-damaging inflammatory microenvironment.

At the signaling level, pro-inflammatory cytokines including IL-6 and IL-1β exert pro-inflammatory effects by activating the Janus kinase/signal transducer and activator of transcription (JAK/STAT) pathway. Upon IL-6 binding to its receptor, the gp130 co-receptor activates JAK1/JAK2, which further phosphorylates STAT3. Subsequently, phosphorylated STAT3 translocates into the nucleus to drive the transcription of pro-inflammatory target genes (Lv et al., 2024). Excessive activation of the JAK/STAT pathway has been confirmed as a core signaling cascade underlying the cytokine storm in acute COVID-19 (Grant et al., 2021). In PCS, persistent secretion of IL-6 and other cytokines suggests that this pathway may remain chronically and low-level activated (Aid et al., 2026), serving as one of the molecular bases for sustained inflammation.

Within the above chronic inflammatory milieu, persistent low-grade activation of the NLRP3 inflammasome acts as a critical node for amplifying inflammatory signaling. SARS-CoV-2 directly activates the NLRP3 inflammasome through multiple mechanisms. Viral ORF3a and E proteins induce ionic imbalance; ORF3a, ORF7a, and NSP16 cause lysosomal dysfunction; ORF7b promotes NLRP3 inflammasome assembly; and the N protein directly triggers this pathway. In PCS, multiple danger-associated molecular patterns, including mitochondrial DNA (mtDNA) leakage, reactive oxygen species (ROS) accumulation and K^+^ efflux, synergistically maintain NLRP3 activation, leading to continuous production of IL-1β and IL-18 and further driving chronic inflammation (He et al., 2024).

Moreover, chronic inflammation is not restricted to the circulatory system. In patients, CD3/CD8 T lymphocytes and CD68 macrophages infiltrate the skeletal muscle, and some of the inflammatory infiltration is dominated by macrophages (Hejbøl et al., 2022). Such local immune infiltration, together with systemic cytokine release, forms a vicious cycle that exacerbates inflammatory responses and contributes to persistent fatigue and impaired tissue repair. Once an excessive inflammatory state is established, specific signals released by damaged tissues may self-maintain this condition, which represents one of the key mechanisms accounting for lingering PCS symptoms after viral clearance.

Notably, there remains academic debate over whether chronic inflammation in PCS necessarily relies on viral persistence. Accumulating evidence suggests that even after complete viral clearance, immune system reprogramming triggered by initial infection alone can independently sustain chronic inflammation. SARS-CoV-2 is capable of reprogramming hematopoietic stem and progenitor cells to induce long-term alterations in innate immunity (Cheong et al., 2023; Zolotarenko et al., 2025). Nevertheless, other studies confirm that persistent viral RNA or proteins residing in tissues including the gut and lungs act as key sources of antigenic stimulation (Gareau and Barrett, 2023; Zuo et al., 2024). These two viewpoints are not mutually exclusive. An integrated framework posits that viral persistence and immune epigenetic reprogramming may play dominant roles with varying weights in distinct patient subgroups, or sequentially dominate disease progression at different stages within a single patient. As clearly stated in the review by Liu et al., complex interactions exist between viral persistence and other etiological hypotheses (Liu S. et al., 2025). Such complexity dictates that chronic inflammation in PCS cannot be simply attributed to one single driver, and explains why interventions targeting a single pathway exhibit markedly variable efficacy across patients. Further longitudinal studies are still needed to validate the relevant controversies.

### 2.3 Mitochondrial dysfunction and energy metabolism

Mitochondrial dysfunction serves as a core pathological mechanism underlying the onset, progression and persistence of PCS. SARS-CoV-2 induces sustained abnormalities in mitochondrial structure and function through multiple pathways, including direct mitochondrial attack, amplified oxidative stress, collapsed energy metabolism, immune dysregulation and multisystem injury, ultimately leading to the long-term persistence of core symptoms such as fatigue.

A growing body of evidence indicates that mitochondrial dysfunction acts as a pivotal pathological link between SARS-CoV-2 infection and PCS. Via its spike protein, membrane protein, and accessory proteins including ORF3a, ORF5, ORF6, and ORF9b, SARS-CoV-2 directly targets mitochondria, disrupts the outer membrane structure, inhibits mitochondrial antiviral signaling protein signaling, interferes with the function of mitochondrial electron transport chain complexes I, III and V, and reduces mitochondrial membrane potential. These alterations result in structural mitochondrial damage and impaired energy synthesis capacity (Lee et al., 2025). Such direct injury persists even after the resolution of acute infection, laying the initial foundation for persistent mitochondrial dysfunction. For instance, ORF3a forms ion channels on the mitochondrial outer membrane, disturbs calcium homeostasis and triggers apoptosis, whereas ORF6 remodels the mitochondrial proteome and suppresses mitochondrial antiviral signaling protein-mediated signaling (De Angelis et al., 2023). The E protein is mainly localized to the endoplasmic reticulum/ERGIC/Golgi membranes, disrupts endoplasmic reticulum calcium stores, and indirectly impairs endoplasmic reticulum–mitochondrial calcium trafficking, thereby precipitating mitochondrial dysfunction (Bhowal et al., 2023). Multiple clinical studies have detected abnormal mitochondrial respiratory function, reduced ATP production, dysregulated mitochondrial gene expression, and mtDNA damage in peripheral blood monocytes, skeletal muscle and brain tissues of long COVID patients, further confirming that direct viral assault induces persistent mitochondrial injury (Streng et al., 2022).

On this basis, electron leakage from the electron transport chain of damaged mitochondria is increased, leading to massive production of mitochondrial reactive oxygen species and subsequent severe oxidative stress. Studies have shown that oxidative stress markers such as F2-isoprostanes and malondialdehyde are markedly elevated in long COVID patients, while the levels of antioxidants including coenzyme Q10, superoxide dismutase and glutathione are significantly decreased, indicating sustained oxidative stress imbalance (Stufano et al., 2023). Excessive mitochondrial reactive oxygen species further attacks mitochondrial DNA, lipid membranes and respiratory chain proteins, exacerbating mitochondrial damage and forming a vicious cycle. Meanwhile, mitophagy of damaged mitochondria is suppressed with impaired function of the PINK1/Parkin pathway. Damaged mitochondria cannot be efficiently cleared, continuously releasing ROS and mtDNA, which further activate the NLRP3 inflammasome and NF-κB pathways and drive chronic low-grade inflammation (Lee et al., 2025).

Mitochondrial dysfunction directly reduces the efficiency of oxidative phosphorylation and leads to insufficient ATP generation, representing the most immediate biochemical mechanism of PCS. The ATP content in skeletal muscle and brain tissues of PCS patients is significantly reduced and closely correlated with fatigue severity. Insufficient energy supply in high-energy-consuming organs directly contributes to persistent fatigue, exercise intolerance and post-exertional symptom exacerbation. Meanwhile, energy deficiency enhances compensatory glycolysis and lactate accumulation, further aggravating myalgia and general fatigue. Magnetic resonance spectroscopy studies have confirmed that the recovery time of skeletal muscle phosphocreatine is markedly prolonged in PCS patients, reflecting underlying mitochondrial energy metabolic disorders (Chen L.-z. et al., 2025).

Molnar et al. further summarized that mitochondrial dysfunction triggers cellular energy deficiency, oxidative stress, immune dysregulation and metabolic disorders, which collectively contribute to PCS (Molnar et al., 2024). Notably, this review highlighted that mitochondrial dysfunction in PCS shares pathological features with other chronic conditions such as aging and HIV infection, including impaired oxidative phosphorylation, elevated ROS production and dysregulated mitophagy. This comparative perspective indicates that insights gained from other mitochondrial-related diseases can inform the understanding and therapeutic development of PCS.

### 2.4 RAS imbalance

In addition to the immune-mitochondrial axis, the long-term symptoms experienced by some PCS patients may result from RAS imbalance (Shetty et al., 2021). The RAS is an essential humoral regulatory system composed of a series of peptide hormones and corresponding enzymes. Its primary physiological functions are to regulate and maintain blood pressure, water and electrolyte homeostasis, and preserve the relative stability of the internal environment. RAS imbalance adversely affects the function of multiple organ systems.

Angiotensin II (Ang II) is the most representative bioactive peptide within the RAS (Ni et al., 2020). Under physiological conditions, ACE2 catalyzes the conversion of Ang II to angiotensin 1–7 (Ang 1–7). Ang 1-7 binds to the Mas receptor and exerts vasodilatory, vascular-protective, anti-fibrotic, anti-proliferative, and anti-inflammatory effects. SARS-CoV-2 infection is accompanied by downregulation of ACE2 expression, a mechanism involving a disintegrin and metalloproteinase 17 (ADAM17). The binding of the SARS-CoV-2 spike protein to ACE2 activates the protease activity of ADAM17, which cleaves the extracellular domain of membrane-bound ACE2, leading to ACE2 shedding and loss of enzymatic activity. ADAM17 activation exerts dual pro-inflammatory effects. On the one hand, ACE2 shedding reduces the conversion of Ang II to Ang 1-7, disrupting the protective arm of the RAS. On the other hand, ADAM17 cleaves membrane-bound TNF-α precursors to release soluble TNF-α, which directly exacerbates inflammation. In addition, ADAM17 can also cleave the IL-6 receptor to generate soluble IL-6R, amplifying the pro-inflammatory effects of IL-6 via trans-signaling pathways (Garbers et al., 2011; Zipeto et al., 2020; Healy, 2022).

**FIGURE 3:**
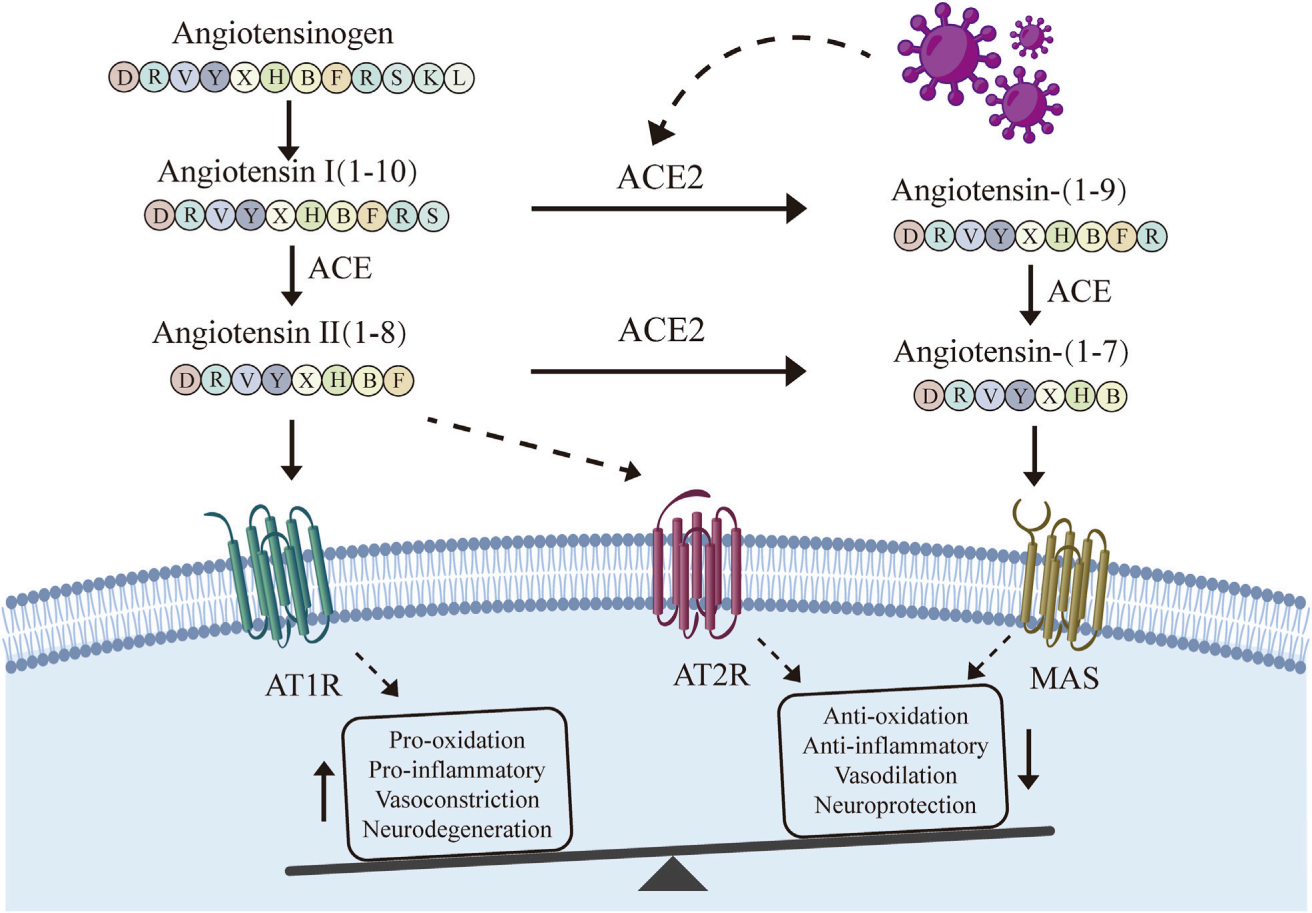
RAS imbalance leads to PCS. ACE2 converts Ang II into Ang 1-7, which binds to MAS receptors and exerts vasodilatory, protective, and other effects. SARS-CoV-2 infection reduces ACE2 expression, causing elevated Ang II and hyperactivation of the RAS, which leads to vasoconstriction, hypertrophy, and other effects, including organ damage such as pulmonary edema and acute kidney injury, which can lead to PCS.

Decreased ACE2 expression elevates Ang II levels and induces excessive RAS activation (Silhol et al., 2020). Upon binding to angiotensin type 1 receptor (AT1R), Ang II triggers vasoconstriction, hypertrophy, fibrosis, proliferation, inflammation, and oxidative stress (El-Arif et al., 2021). This cascade reaction causes tissue damage in multiple organs, including pulmonary edema, acute kidney injury, myocardial hypertrophy, and interstitial fibrosis; such injuries may persist into the chronic phase. Due to incomplete tissue repair, sustained ACE2 downregulation maintains excessive RAS activation even after viral clearance, thereby contributing to the development of PCS. The mechanistic role of RAS imbalance in PCS is illustrated in Figure 3. Notably, current studies on the RAS mechanism in PCS still have considerable limitations. Most existing researches are cross-sectional observational studies and lack long-term longitudinal follow-up data. Furthermore, the majority of studies fail to adequately control for confounding factors, including underlying cardiovascular diseases, antihypertensive medications, and obesity. PCS patients with comorbid hypertension or diabetes already exhibit inherent RAS imbalance, making it difficult to distinguish whether RAS abnormalities represent a continuation of pre-existing changes caused by primary diseases or an independent pathological process induced by SARS-CoV-2. Therefore, such RAS alterations cannot be solely attributed to SARS-CoV-2 infection. These limitations suggest that the potential of RAS as a therapeutic target for PCS requires further validation in rigorously designed prospective cohort studies.

### 2.5 Gut microbial dysbiosis and MGB axis disturbance

A growing body of evidence suggests that the pathogenesis of PCS is closely associated with gut microbiota. A considerable proportion of patients with long COVID exhibit alterations in the gut microbiome, characterized by reduced microbial diversity, depletion of Short-chain fatty acids (SCFAs)-producing taxa, and enrichment of opportunistic or pro-inflammatory microorganisms (Liu et al., 2022a; Caliman-Sturdza et al., 2025; Iqbal et al., 2025). Emerging studies have indicated that SARS-CoV-2 may induce neurological dysfunction via the MGB axis. SARS-CoV-2 can still be detected in small intestinal biopsy samples obtained from patients 1 year after symptom onset (Gareau and Barrett, 2023). Viral infection disrupts the composition of the gut microbial community and impairs intestinal barrier function, subsequently triggering central nervous system damage through the MGB axis. Studies have shown that insufficient immunomodulatory microbiota, including *Faecalibacterium prausnitzii*, *Eubacterium rectale*, and *Bifidobacterium adolescentis*, persist in the gut even 30 days after viral clearance, leading to elevated concentrations of TNF-α, CXCL10, CCL2, and IL-10 and provoking inflammatory responses (Yeoh et al., 2021). Intestinal inflammation is a prominent feature of altered MGB axis signaling, which induces neuroinflammation in the brain and consequently contributes to cognitive impairment and brain fog in PCS (Gareau and Barrett, 2023). As a key butyrate-producing commensal bacterium, *Faecalibacterium prausnitzii* is widely recognized as an anti-inflammatory microbe. It exerts multiple beneficial effects, including anti-inflammation, immune regulation, maintenance of cognitive memory, and preservation of lysozyme activity (Yeoh et al., 2021). This bacterium is critical for sustaining cognitive homeostasis through the MGB axis; its depletion weakens anti-inflammatory defenses, sustains chronic inflammation, and ultimately facilitates the development of PCS.

**FIGURE 4:**
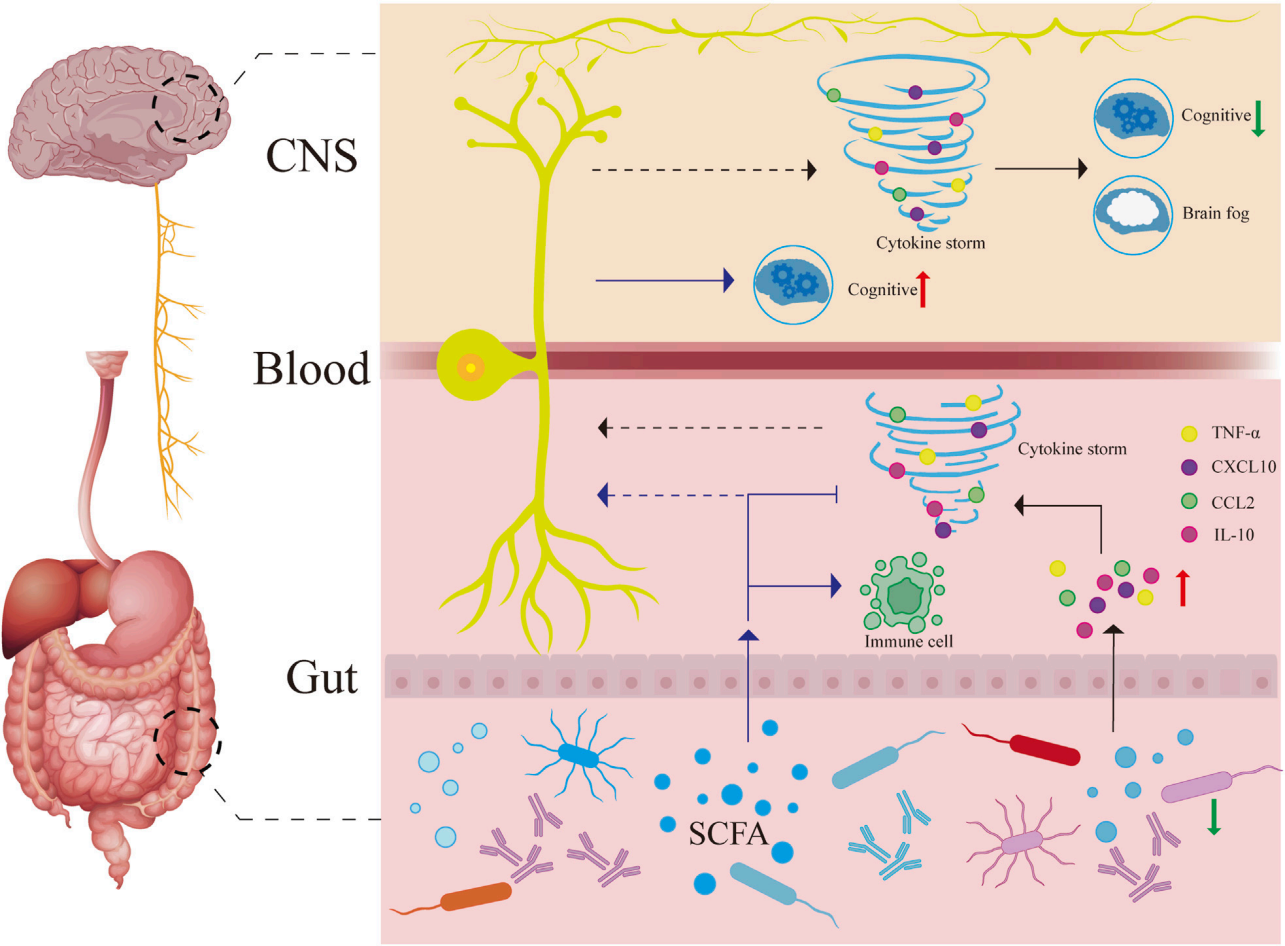
Influence of gut microbes on PCS. SARS-CoV-2 can cause neurological damage through the MGB axis, and insufficient immune flora in the gut leads to increased concentrations of inflammatory factors such as TNF-α, which in turn leads to PCS and brain fog symptoms; when SARS-CoV-2 infects the cells, the S protein binds to the ACE2 receptor affecting tryptophan uptake in the small intestine, and in addition, leads to an increase in the activation of the KP, which causes a decrease in tryptophan leading to PCS.

SARS-CoV-2 also disrupts intestinal homeostasis via ACE2. ACE2 binds to amino acid transporters and plays an essential role in intestinal amino acid absorption (Hashimoto et al., 2012). Occupation of ACE2 by SARS-CoV-2 impedes tryptophan absorption in small intestinal epithelial cells, thereby reducing the synthesis of antimicrobial peptides in Paneth cells and rendering the host susceptible to intestinal inflammation. Meanwhile, the virus disturbs the homeostatic interplay between the microbiota and intestinal immune system, leading to intestinal inflammatory disorders, impaired intestinal energy absorption, and ultimately the onset of PCS. Furthermore, tryptophan is an essential amino acid primarily degraded via the kynurenine pathway (KP) (Eroğlu et al., 2021). SARS-CoV-2 infection enhances KP activation. Accumulating evidence demonstrates that elevated cerebral KP metabolites induce neurotoxicity, contributing to central fatigue and memory impairment. In addition, tryptophan serves as a precursor for melatonin synthesis; reduced tryptophan availability decreases melatonin levels, thereby disturbing sleep regulation and immune function and exacerbating fatigue (Huang et al., 2023). The impacts of gut microbial dysbiosis and disrupted MGB axis on PCS are illustrated in Figure 4. Given the slow recovery of the gut microbial ecosystem, microbiota imbalance and associated mild intestinal inflammation may persist for months, sustaining PCS symptoms long after the resolution of acute viral infection.

Collectively, these four pathological processes do not act independently and in parallel in PCS pathogenesis, but interact reciprocally to form a self-sustaining vicious cycle. Future therapeutic strategies may shift toward multi-target combination therapy rather than complete inhibition of a single target. In summary, the excessive inflammatory state driven primarily by immune dysregulation, together with mitochondrial dysfunction, RAS imbalance, and gut microbial dysbiosis, results in multi-tissue injury. Since these upstream driving mechanisms can persist independently of residual viral presence, this explains why PCS symptoms may emerge or worsen even after antiviral treatment, and why clinical recovery from PCS is often protracted.

## 3 Potential therapeutic targets and pathways

### 3.1 Potential therapeutic targets for immune dysregulation and chronic inflammation

#### 3.1.1 TLR4/TLR7

The excessive and self-sustaining inflammatory state in PCS is partially maintained by persistent activation of the innate immune system. Toll-like receptor 4 (TLR4) and Toll-like receptor 7 (TLR7) are key pattern recognition receptors that detect pathogen-associated molecular patterns and damage-associated molecular patterns (DAMPs) in innate immunity (Poulas et al., 2020). They also serve as critical upstream sensors linking SARS-CoV-2 infection to immune dysregulation and chronic inflammation in PCS. Upon SARS-CoV-2 invasion, the spike protein can bind to multiple TLRs, thereby activating innate immune cells such as macrophages and dendritic cells. Among these receptors, TLR4 and TLR7 are the most potent inducers of pro-inflammatory responses (Dahpy et al., 2026).

TLR4 is localized on the cell surface and primarily recognizes endogenous DAMPs, including high-mobility group box 1 and S100 proteins. By contrast, TLR7 resides on the endolysosomal membrane and specifically senses single-stranded RNA. During acute infection, both receptors are directly activated by viral spike protein and viral RNA, driving the expression of pro-inflammatory cytokines such as interleukin-6 (IL-6), tumor necrosis factor-α (TNF-α), and interleukin-1β (IL-1β), as well as type I interferons via the myeloid differentiation primary response 88 dependent pathway (Salvi et al., 2021; Holms, 2022; Sahanic et al., 2023). In the PCS stage, even after viral clearance, persistently elevated endogenous DAMPs and residual viral RNA can sustain chronic activation of TLR4 and TLR7, rendering them crucial upstream nodes driving immune dysregulation and persistent chronic inflammation.

Accumulating evidence has demonstrated persistent functional dysregulation of the TLR4 and TLR7 pathways in PCS patients. Ghorra et al. (2024) performed *in vitro* TLR pathway stimulation assays using whole blood samples from 50 PCS patients and 25 healthy controls. The results showed that following stimulation of the TLR4 and TLR7/8 pathways, the levels of interleukin-1α (IL-1α), IL-1β, interferon-α (IFNα), interferon-γ (IFNγ), and TNF-α in PCS patients were significantly lower than those in healthy individuals. This hyporesponsiveness does not indicate diminished TLR pathway function; instead, it reflects a chronic basally activated immune state that leads to exhausted responsiveness to further stimulation (Ghorra et al., 2024).

Furthermore, Holms (2022) proposed a self-sustaining model of persistent TLR4 activation in long COVID. Excessive activation of the TLR4/RAGE axis by the SARS-CoV-2 spike protein during acute infection induces sustained upregulation of S100A8/A9 as endogenous DAMPs even after viral clearance. S100A8/A9 continuously binds to TLR4/RAGE to maintain long-term expression of IL-1β, IL-6, and TNF-α, forming a pathological self-sustaining inflammatory feedback loop (Holms, 2022). These findings highlight the vital role of the TLR4 and TLR7/8 pathways in the pathophysiology of PCS.

Therefore, TLR4 and TLR7 represent important upstream intervention targets linking initial SARS-CoV-2 infection to the persistence of immune dysregulation and chronic inflammation in PCS. Selective or combined antagonism of TLR4/TLR7 can block inflammatory signals triggered by DAMPs and residual viral RNA at the source, thereby reducing the chronic inflammatory burden of PCS. Nevertheless, it should be noted that TLRs are indispensable for host defense; long-term systemic inhibition of TLR4 or TLR7 may increase the risk of secondary infection. In addition, clinical trial data on specific TLR antagonists in PCS patients are currently lacking.

#### 3.1.2 JAK/STAT pathway

The JAK-STAT signaling pathway serves as a core hub for the signal transduction of multiple pro-inflammatory cytokines, including IFN-γ, IL-6, IL-1β, and TNF-α. This pathway exhibits persistent activation in patients with PCS, laying the foundation for chronic inflammation and immune exhaustion (Aid et al., 2026). In addition, chronically secreted IL-6 and IL-1β from exhausted T cells exert pro-inflammatory effects predominantly through the JAK/STAT pathway. The binding of IL-6 to its receptor recruits glycoprotein 130, leading to the activation of JAK1 and JAK2. Activated JAKs subsequently phosphorylate STAT3. Phosphorylated STAT3 undergoes dimerization and translocates into the nucleus, where it drives the transcription of pro-inflammatory and pro-survival genes (Xu et al., 2025). Accumulating evidence indicates that the COVID-19 cytokine storm is closely associated with excessive JAK/STAT activation (Rodriguez and Carnevale, 2024). In PCS patients, persistently low-level elevation of IL-6 suggests chronic, subacute hyperactivity of the JAK/STAT pathway (Aid et al., 2026).

Transcriptomic analysis of peripheral blood mononuclear cells from COVID-19 convalescents reveals that STAT3 target genes remain upregulated for months after recovery (Fineschi et al., 2025). Meanwhile, elevated levels of soluble IL-6 receptor in convalescent plasma indicate sustained activation of JAK/STAT-mediated IL-6 trans-signaling, which further amplifies inflammatory responses (Lokau et al., 2025). PCS is characterized by enhanced persistent pro-inflammatory cytokine signaling, complement system activation, and adaptive immune cell exhaustion, indicating that the JAK/STAT pathway is a critical potential therapeutic target. Selective inhibition of JAK1/JAK2 can break the cytokine-driven pro-inflammatory feedforward loop, alleviate fatigue, and improve systemic symptoms of PCS (Marconi et al., 2021; Aid et al., 2026).

#### 3.1.3 NLRP3 inflammasome

The NLRP3 inflammasome is a central cytosolic sensor of the innate immune system and a key molecular hub linking immune dysregulation, mitochondrial damage, and chronic inflammation. Upon activation, the NLRP3 inflammasome catalyzes the cleavage of pro-IL-1β and pro-IL-18 into their mature forms. It also induces pyroptosis by cleaving gasdermin D, thereby releasing abundant DAMPs and amplifying the inflammatory cascade (Cabral et al., 2025). In PCS, multiple persistent danger signals, including mtDNA leakage, excessive ROS production, and K^+^ efflux, collectively sustain NLRP3 inflammasome activation. This renders the NLRP3 inflammasome a core driver of persistent low-grade inflammation (He et al., 2024), which remains active long after acute infection and sustains PCS symptoms.

Existing evidence has confirmed persistent NLRP3 inflammasome activation in PCS patients. Zhang et al. enrolled 161 long COVID patients and detected serum biomarkers 6–9 months after acute infection. The results showed that the levels of NLRP3, caspase-1, IL-18, IL-1β, and gasdermin D were significantly elevated and positively correlated with the severity of acute infection. Their findings indicated that long COVID severity is closely associated with systemic inflammatory responses and NLRP3 activation, suggesting NLRP3 as a promising therapeutic target for PCS (Zhang et al., 2026).

Therefore, the NLRP3 inflammasome represents a critical intervention target linking mitochondrial oxidative stress, immune dysregulation, and chronic inflammation in PCS. Direct NLRP3 inhibition can block the maturation of IL-1β and IL-18 and interrupt the inflammatory amplification loop (Cabral et al., 2025), which may contribute to PCS treatment. A fundamental issue remains whether complete NLRP3 inhibition is the optimal strategy, or whether context-dependent modulation of its activity yields superior therapeutic outcomes. Long-term NLRP3 suppression may weaken host defense and produce negative effects (Zheng et al., 2020). Furthermore, potential crosstalk among different inflammasomes should be considered. Inhibition of NLRP3 may lead to compensatory activation of other pathways such as AIM2, NLRC4, and cGAS, thereby altering immune responses in an unpredictable manner.

### 3.2 Potential therapeutic targets for mitochondrial dysfunction

#### 3.2.1 AMPK/NRF2 signaling pathway

The AMPK/NRF2 axis acts as a core signaling cascade linking energy metabolism, oxidative stress defense, and inflammatory regulation. It also serves as a critical intervention node for the vicious cycle between mitochondrial dysfunction and chronic inflammation in PCS. As a central regulator of cellular energy homeostasis, adenosine monophosphate-activated protein kinase (AMPK) is activated under energy stress conditions, such as decreased ATP levels and an elevated AMP/ATP ratio. Activated AMPK promotes the dissociation and nuclear translocation of nuclear factor erythroid 2-related factor 2 (NRF2) from its cytoplasmic repressor Kelch-like ECH-associated protein 1 (KEAP1). This occurs through direct phosphorylation of NRF2 and inhibition of KEAP1 (Joo et al., 2016; Huang et al., 2024). Upon nuclear entry, NRF2 binds to antioxidant response elements and drives the expression of a panel of antioxidant enzymes and cytoprotective proteins, including superoxide dismutase, catalase, and heme oxygenase-1, thereby restoring cellular redox homeostasis (Taguchi et al., 2011; Ma, 2013).

In PCS patients, mitochondrial dysfunction leads to excessive ROS production, while impairment of the AMPK/NRF2 pathway renders cells incapable of effectively coping with this oxidative stress burden. Qu et al. demonstrated that SARS-CoV-2 infection markedly downregulates NRF2 protein levels and the expression of NRF2-dependent genes in human bronchial epithelial cells and mouse lung tissues. This inhibitory effect is also observed in variants such as Omicron, which has been identified as a potent NRF2 inhibitor. Notably, Nrf2-deficient mice exhibit exacerbated clinical manifestations and intensified pulmonary inflammation after infection, confirming the protective role of NRF2 during viral infection (Qu et al., 2023). Existing studies have also revealed that the SARS-CoV-2 non-structural protein NSP14 enables viral evasion of host antioxidant defenses by targeting the NRF2/HO-1 axis, further clarifying the molecular mechanism underlying NRF2 suppression during infection (Zhang S. et al., 2022). In addition, skeletal muscle biopsies from PCS patients have revealed cytochrome c oxidase-negative fibers and abnormal mitochondrial ultrastructure (Hejbøl et al., 2022), indicating insufficient clearance of oxidative damage. These findings are highly consistent with inadequate NRF2-mediated antioxidant defense, suggesting that NRF2 acts as a key effector target.

Accordingly, restoring AMPK/NRF2 axis activity can simultaneously correct energy metabolic defects, eliminate oxidative damage, and suppress inflammatory amplification. This improves systemic inflammatory status and alleviates PCS symptoms. At present, most studies on NRF2 activation focus on acute lung injury or cellular models, and the comprehensive beneficial effects of NRF2 activation on multi-organ injury in PCS remain unclear. Furthermore, whether long-term NRF2 activation may interfere with physiological ROS signaling or induce off-target effects still requires further validation.

#### 3.2.2 PINK1/Parkin pathway

The PINK1/Parkin pathway serves as the core quality-control system for the selective clearance of damaged mitochondria. Upon mitochondrial damage and loss of membrane potential, PINK1 stably accumulates on the outer mitochondrial membrane, becomes activated via autophosphorylation, and recruits the E3 ubiquitin ligase Parkin. Activated Parkin mediates the ubiquitination of outer mitochondrial membrane proteins. These ubiquitin chains are recognized by autophagy receptors, ultimately sequestering damaged mitochondria into autophagosomes for lysosomal degradation. This quality-control mechanism ensures the timely elimination of dysfunctional mitochondria, preventing the accumulation of damaged organelles and the sustained release of pro-inflammatory molecules such as ROS and mtDNA (Agarwal and Muqit, 2022; Narendra and Youle, 2024).

In PCS, PINK1/Parkin-dependent mitophagy is markedly suppressed. This abnormality is closely associated with SARS-CoV-2 hijacking of mitochondria, ORF9b-induced mtDNA leakage, and excessive ROS production caused by impaired oxidative phosphorylation. Existing evidence indicates that persistent inflammation and metabolic reprogramming during acute and post-acute SARS-CoV-2 infection inhibit PINK1/Parkin-mediated mitophagy, facilitating the accumulation of dysfunctional mitochondria and the maintenance of long-term PCS symptoms (Delpino and Quarleri, 2025). Meanwhile, SARS-CoV-2 can hinder the binding of the autophagy receptor p62 to LC3, further blocking the selective enclosure of damaged mitochondria into autophagosomes (Shang et al., 2021). Furthermore, Zhou et al. reported that Parkin can induce ubiquitination and degradation of the SARS-CoV-2 main protease to restrict viral replication. However, viral infection itself downregulates Parkin expression in lung tissues, implying that SARS-CoV-2 possesses a mechanism to evade this host defense (Zhou et al., 2024). Notably, Ali et al. detected the expression of mitophagy-related genes in peripheral blood mononuclear cells from PCS patients 10 months post-infection, and found that Parkin expression was significantly upregulated compared with convalescent controls; meanwhile, Nrf1 and Drp1 levels were also markedly elevated (Ali et al., 2026). Such upregulated gene expression indicates a compensatory host response to mitochondrial injury, yet downstream autophagic pathways remain functionally restricted. The clearance efficiency of damaged mitochondria remains insufficient, resulting in persistent release of ROS and mtDNA. Collectively, these pathogenic processes block the clearance of damaged mitochondria and sustain excessive ROS generation, thereby forming a self-sustaining vicious cycle.

Accordingly, the PINK1/Parkin axis acts as a vital intervention target linking defective mitochondrial quality control and chronic inflammation in PCS. Enhancing PINK1/Parkin-mediated mitophagy facilitates the removal of damaged mitochondria, reduces the production of ROS and mtDNA, and further blocks the persistent activation of downstream NLRP3 inflammasome and cGAS-STING pathways. This breaks the vicious cycle bridging mitochondrial dysfunction and inflammation. At present, several novel PINK1 and Parkin activators can lower the activation threshold of mitophagy and have shown therapeutic potential in related mitochondrial disease models (Rosencrans et al., 2025). Nevertheless, their applicability and efficacy in PCS remain to be further investigated.

### 3.3 Potential therapeutic targets for RAS imbalance

#### 3.3.1 ACE2/Ang 1–7/Mas protective axis

The ACE2/Ang 1–7/Mas axis is an endogenous negative regulatory pathway of the classical RAS and physiologically counteracts the pro-inflammatory ACE/Ang II/AT1R axis. Under normal conditions, ACE2 catalyzes the conversion of Ang II into Ang 1-7, which binds to the Mas receptor and exerts protective effects including vasodilation, anti-inflammation, anti-fibrosis and anti-proliferation (Li et al., 2017; Santos et al., 2017). SARS-CoV-2 infection causes substantial consumption of ACE2, leading to reduced Ang 1-7 generation and impaired Ang II degradation, thereby severely weakening the function of this protective axis. The Ang 1–7/Ang II ratio is a key factor driving disrupted RAS homeostasis (Ghimire et al., 2023). Therefore, restoring the activity of the ACE2/Ang 1–7/Mas axis can serve as a strategy to correct RAS imbalance.

Existing studies have shown that COVID-19 is characterized by decreased levels of ACE2 and Ang 1-7, which may play a central role in the devastating cytokine storm of the disease. ACE2 supplementation and Ang 1-7 administration were also tested as therapeutic options in the clinical management of severe COVID-19 cases (Heyman et al., 2021). Sansoè et al. indicated that recombinant human ACE2 mimics endogenous ACE2 activity: on the one hand, it degrades excessive Ang II to produce Ang 1–7; on the other hand, it acts as a decoy receptor to block the binding of SARS-CoV-2 to cellular ACE2. Theoretically, it exerts dual antiviral and anti-inflammatory effects, although clinical outcomes remain inconsistent (Sansoè and Aragno, 2023).

Complex signaling crosstalk exists among various RAS targets, and the prolonged clinical course of PCS poses important challenges to the intervention of the ACE2/Ang 1-7 pathway. In addition, the short half-life of Ang 1-7 itself and its injection administration route limit its long-term application, and Mas receptor agonists are being explored as small-molecule alternatives. Notably, Heyman et al. clearly pointed out that activation of the ACE2/Ang 1–7/MasR axis may even accelerate renal injury under certain conditions, and such contradictory findings need to be further clarified (Heyman et al., 2021). Overall, therapeutic targeting of this axis for PCS intervention represents a field with both challenges and opportunities.

#### 3.3.2 ADAM17 inhibition

ADAM17, also known as tumor necrosis factor-α converting enzyme, is a key transmembrane protease linking RAS imbalance and chronic inflammation in PCS. As described in Section 2.4, the SARS-CoV-2 spike protein activates ADAM17, leading to ACE2 shedding and the release of soluble TNF-α and soluble interleukin-6 receptor. This process drives a vicious cycle of RAS imbalance and inflammatory amplification. Following viral clearance, persistent tissue injury and low-grade inflammation may sustain ADAM17 activity. Accordingly, ADAM17 represents a promising therapeutic target bridging RAS imbalance and chronic inflammation in PCS.

The therapeutic potential of ADAM17 has been preliminarily validated in preclinical COVID-19 studies. Pereira et al. demonstrated that targeting iRhom2/ADAM17 attenuates pseudovirus-induced TNF-α release, and TAPI-0 exerts inhibitory effects in a concentration-dependent manner in human bronchial epithelial cells (Pereira et al., 2024). Studies have also shown that the selective ADAM17 inhibitor JG26 restricts SARS-CoV-2 spread by reducing the availability of soluble ACE2, showing promising antiviral activity (Gentili et al., 2025).

Nevertheless, ADAM17 plays a dual role in host defense, which constitutes a core dilemma for targeting this molecule. Administration of the ADAM17 neutralizing antibody MEDI3622 in K18-hACE2 transgenic COVID-19 mice reduced inflammation and mortality but increased viral load in lung tissues (Hedges et al., 2022). Some PCS patients may harbor persistent low-level residual virus or viral proteins. Whether ADAM17 inhibition interferes with the clearance of residual pathogens and thereby exacerbates or prolongs clinical symptoms remains unsupported by available clinical data. Furthermore, no ADAM17 inhibitors have been approved for clinical use to date (Gentili et al., 2025). This is largely attributed to the high homology of active sites among other ADAM and MMP family members, making it extremely challenging to develop selective ligands and avoid off-target toxicity. The safety risks associated with non-selective inhibition become more prominent in the long-term management of PCS. Currently, no ADAM17 inhibitors have entered PCS-related clinical trials, and this target remains at the preclinical proof-of-concept stage.

### 3.4 MGB axis-related therapeutic targets

#### 3.4.1 IDO1/TDO2

SARS-CoV-2 reduces intestinal tryptophan uptake by occupying intestinal ACE2, while pro-inflammatory cytokines induce Indoleamine 2,3-dioxygenase 1 (IDO1) expression. Together, these two effects drive excessive activation of the KP. IDO1 and tryptophan 2,3-dioxygenase (TDO2) are the rate-limiting enzymes of the KP, and their excessive activation is a key link between gut microbiota dysbiosis and the accumulation of neurotoxic metabolites leading to cognitive impairment. IDO1 is strongly induced by pro-inflammatory cytokines such as IFN-γ, whereas TDO2 is regulated by cortisol. Both enzymes convert tryptophan into kynurenine. Downstream metabolites including 3-hydroxykynurenine and quinolinic acid exert neurotoxic effects, inducing synaptic dysfunction through excessive NMDA receptor activation and oxidative stress (Fesharaki Zadeh et al., 2023).

Existing studies indicate that tryptophan metabolism via the KP is one of the major pathways affected by COVID-19 infection (Thomas et al., 2020; Badawy, 2023). Badawy systematically reviewed 16 studies and confirmed that virus-induced KP activation is predominantly mediated by IDO1, while cortisol further upregulates TDO2 expression and enhances KP flux (Badawy, 2023). The ADAPT longitudinal study followed 128 patients for 12 months and found that persistent KP activation is closely associated with mild cognitive impairment, with limited spontaneous improvement within 12 months. The study highlighted that the KP serves as a potential diagnostic and monitoring biomarker, as well as a promising therapeutic target for brain fog in PCS (Cysique et al., 2023). Sustained KP activation can be detected at 2, 4, and 12 months post-infection, suggesting that IDO1 acts as the major driver of KP activation during the chronic stage.

Inhibition of IDO1/TDO2 can block the production of neurotoxic metabolites and redirect tryptophan toward serotonin and melatonin synthesis. N-acetylcysteine combined with guanfacine has shown preliminary improvements in working memory and executive function in 12 PCS patients (Fesharaki Zadeh et al., 2023). Nevertheless, targeting this pathway faces notable challenges, and currently available IDO1 inhibitors carry potential hepatotoxicity risks. Combination strategies pairing IDO1 inhibitors with JAK inhibitors to attenuate IFN-γ-driven KP activation, or with AhR modulators, may represent more promising approaches. However, supporting safety and efficacy data for such combined regimens remains lacking to date.

#### 3.4.2 SCFAs

SCFAs are pivotal metabolites produced by gut microbiota during the fermentation of dietary fiber and serve as core effector molecules of the MGB axis (Majumdar et al., 2023). Existing evidence indicates a sustained depletion of SCFA-producing bacteria in PCS patients. Patients with PCS consistently exhibit reduced microbial diversity and depletion of beneficial SCFA-producing species, including *Faecalibacterium prausnitzii* and *Bifidobacterium* spp., accompanied by the enrichment of pro-inflammatory taxa such as *Ruminococcus gnavus* and *Bacteroides vulgatus* (Caliman-Sturdza et al., 2025). Among these, the butyrate-producing species *Bifidobacterium pseudocatenulatum* and *Faecalibacterium prausnitzii* show the strongest negative correlation with PCS symptoms at 6 months. Reduced abundance of SCFA-producing bacteria may indicate an elevated risk of long COVID or delayed recovery (Liu et al., 2022a).

Such gut microbial dysbiosis leads to decreased SCFA concentrations. SCFAs activate anti-inflammatory responses in immune cells and inhibit inflammatory signaling pathways such as NF-κB. They also maintain intestinal barrier integrity by upregulating the expression of tight junction proteins, thereby preventing the entry of intestinal endotoxins and bacteria into the circulation and alleviating local and systemic inflammatory responses (Caliman-Sturdza et al., 2025). In particular, butyrate can enhance specific immunity and suppress autoimmunity by promoting IgA and IgG antibody responses in B cells (Davis et al., 2023). Following SARS-CoV-2 infection, persistent depletion of SCFA-producing bacteria reduces SCFA levels, contributing to prolonged inflammation and PCS symptoms. At present, several small interventional studies have demonstrated that microbiome-targeted therapies can alleviate long COVID symptoms. Some interventions restore the abundance of SCFA-producing bacteria and relieve fatigue and cognitive impairment (Caliman-Sturdza et al., 2025). These preliminary findings collectively suggest that restoring SCFA production may represent a viable strategy for alleviating PCS manifestations. Nevertheless, available evidence remains largely based on observational studies, and the causal relationship between direct SCFA pathway targeting and PCS treatment has not been rigorously established. Most studies have small sample sizes, and standardized protocols for SCFA quantification are still lacking, which remain major challenges in this research field. Furthermore, as gut microbial metabolites, SCFAs exhibit bioavailability and tissue distribution modulated by multiple factors. Further research is required to clarify whether exogenous SCFA supplementation or SCFA-producing strains can effectively reach target organs and exert physiological effects.

## 4 Potential therapeutic drugs

Currently, there is no globally standardized therapeutic regimen for PCS. However, high-quality clinical studies in recent years have gradually identified several agents with definite ameliorative potential. Most of these drugs exert therapeutic effects by regulating chronic inflammation, improving mitochondrial function, alleviating neuropsychiatric symptoms, and correcting immune imbalance, with their clinical benefits mainly focused on improving the core symptom of fatigue. In this article, we classify these drugs into two categories: potential agents based on target mechanisms and agents acting through other mechanisms. This review aims to provide scientific insights for the drug development and clinical translation of PCS.

### 4.1 Potential drugs based on target mechanism

#### 4.1.1 Persistent viral infection

Persistent viral infection is considered the root cause of PCS, which may result from incomplete clearance of SARS-CoV-2. The virus can evade immune elimination and trigger sustained immune responses, exacerbating chronic symptoms through cytokine imbalance, T cell exhaustion, and systemic inflammation. Therefore, reducing and eliminating residual viral reservoirs represents a rational approach for the amelioration and treatment of PCS (Gupta et al., 2025).

As an antiviral agent, Paxlovid (nirmatrelvir/ritonavir) exhibits potential to prevent the progression to PCS when administered during acute infection. A meta-analysis covering 23 studies with more than 150,000 participants found that Paxlovid administration within 5 days of symptom onset during acute COVID-19 was associated with a 62% reduction in PCS risk (Amani and Amani, 2023). Nirmatrelvir acts as a peptidomimetic inhibitor of the main protease of SARS-CoV-2, thereby blocking viral replication. Combined with low-dose ritonavir, the latter slows the metabolism of nirmatrelvir by inhibiting cytochrome P450 3A4. Paxlovid has been approved by the FDA for the treatment of adults with mild-to-moderate COVID-19 who are at high risk of progressing to severe illness (Geng et al., 2024). Nevertheless, current research conclusions remain inconsistent. Some studies have demonstrated that 15-day Paxlovid treatment fails to yield statistically significant improvements in core symptoms such as fatigue and brain fog compared with the placebo group in PCS patients (Zheng et al., 2022). By contrast, a small case report observed a 55.3% improvement in fatigue severity lasting up to 2 years among 14 PCS patients receiving combined antiviral therapy (Pridgen and Putrino, 2026). Such discrepant outcomes may be explained by the pharmacological mechanism of Paxlovid, which primarily targets active viral replication. One prominent hypothesis for PCS pathogenesis is viral persistence, in which SARS-CoV-2 persists in a latent state within specific tissues and forms viral reservoirs. Under such non-replicating conditions, therapeutic strategies mainly targeting viral replication may show limited efficacy in PCS management.

Regrettably, the protective mechanism of oral antiviral agents remains poorly elucidated. Given that severe COVID-19 represents a major risk factor for PCS, the prevailing view suggests that oral antivirals suppress viral replication, attenuate the progression of acute COVID-19, and mitigate acute SARS-CoV-2-induced immune dysregulation, thereby reducing the subsequent risk of PCS development.

#### 4.1.2 Potential drugs targeting immune dysregulation and chronic inflammation

##### 4.1.2.1 TLR4/TLR7 antagonists

Naltrexone is a synthetic opioid receptor antagonist widely used for the treatment of opioid and alcohol dependence (Brown and Panksepp, 2009). Existing studies have found that low-dose naltrexone (LDN) exerts potential therapeutic effects on post-COVID fatigue syndrome through multiple mechanisms at a daily dose no more than 4.5 mg. Its core mechanisms include antagonizing TLR4 expressed on microglia and immune cells, reducing the levels of pro-inflammatory factors such as IL-6 and TNF-α to alleviate neuroinflammation and systemic chronic inflammation, as well as elevating endogenous β-endorphin concentrations to regulate neuroimmune function and modulate pathways associated with central fatigue (Tamariz et al., 2024).

In terms of clinical symptom improvement, LDN markedly relieves the core manifestations of post-COVID fatigue syndrome, including persistent severe fatigue, post-exertional malaise, sleep disturbances and cognitive impairment. It also alleviates accompanying symptoms such as chronic pain and orthostatic intolerance, and improves patients’ daily activity ability and health-related quality of life. With favorable safety and mild side effects, LDN is expected to become an effective intervention strategy for PCS (Naik et al., 2024). Nevertheless, large-scale randomized controlled trials (RCT) investigating LDN for PCS are still ongoing, and the definitive efficacy as well as the optimal dosing regimen remain to be further validated. In addition to naltrexone, other candidate drugs targeting TLR and their mechanisms of action are summarized in Table 1.

**TABLE 1:** Other PCS candidate drugs targeting TLRs.

Drug	Mechanism	References
Ibudilast	Inhibits microglial activation, downregulates pro-inflammatory factors including TNF-α, IL-1β and IL-6, upregulates the anti-inflammatory factor IL-10, and suppresses TLR4-mediated NF-κB and MAPK signaling pathways	Li et al. (2021), Angelopoulou et al. (2022)
Bezisterim	It reduces ERK phosphorylation, inhibits TLR4- and TNF-induced NF-κB activation and downstream signaling, and decreases the expression of pro-inflammatory cytokines and chemokines	Reading et al. (2025)
Enpatoran	It highly selectively inhibits TLR7/8, blocks persistent endosomal TLR7/8 activation triggered by residual viral RNA, and reduces the production of IFN-α and pro-inflammatory cytokines	Klopp-Schulze et al. (2021), Port et al. (2021)
Lodonal	It antagonizes TLR4 to suppress the production of pro-inflammatory cytokines such as TNF-α and IL-6, thereby mitigating persistent fatigue driven by chronic low-grade inflammation	Hsu et al. (2017)
Resatorvid	It selectively binds to the Cys747 residue in the intracellular domain of TLR4, interfering with the interaction between TLR4 and adaptor molecules TIRAP and TRAM. It blocks the activation of downstream NF-κB and MAPK, and inhibits the production of NO, TNF-α and IL-6	Matsunaga et al. (2011), Khomeijani-Farahani et al. (2024)

##### 4.1.2.2 JAK inhibitors

The JAK/STAT signaling pathway is persistently activated in PCS patients, forming the biological basis of chronic inflammation and immune exhaustion. A multiomics study published in *Nature Immunology* in 2025 demonstrated that the hallmark manifestations of long COVID include sustained enhancement of pro-inflammatory cytokine signaling, complement system activation, and adaptive immune cell exhaustion, suggesting that the JAK/STAT pathway serves as a critical potential therapeutic target (Aid et al., 2026). Based on this mechanistic rationale, JAK inhibitors have been repurposed as candidate therapeutic agents for PCS.

Clinical evidence regarding JAK inhibitors for PCS is accumulating rapidly but remains in the early stage overall. Abrocitinib is a selective JAK1 inhibitor approved for the treatment of atopic dermatitis. After identifying abnormalities in inflammatory proteins and signaling pathways from multiomics data, researchers initiated a phase 2a clinical trial. Available preliminary data showed that the improvement rates of fatigue and dyspnea reached 65% and 45% in the abrocitinib group, compared with approximately 45% in the placebo group (Aid et al., 2026). Upadacitinib is a potent and selective JAK inhibitor that blocks the JAK/STAT signaling pathway, suppresses the release of pro-inflammatory cytokines, and mitigates inflammatory responses. Relevant observations indicated that three long COVID patients presenting predominantly with severe neuropsychiatric symptoms exhibited significant reductions in monocyte pro-inflammatory factors TNF-α and IL-1β, as well as compensatory anti-inflammatory factor IL-10 following upadacitinib intervention. Meanwhile, serum kynurenine levels closely related to neuroinflammation and energy metabolic dysfunction decreased synchronously (Jyonouchi et al., 2025).

Although JAK inhibitors have a solid theoretical basis for PCS treatment by targeting the JAK/STAT pathway, their clinical application still faces considerable challenges. Current evidence is mainly derived from small-sample observational studies or case reports, lacking high-level evidence from large-scale RCTs, and both efficacy and safety remain inconclusive.

#### 4.1.3 Potential drugs for improving mitochondrial function and energy metabolism

AXA1125 is a novel endogenous metabolic modulator composed of leucine, isoleucine, valine, arginine, glutamine, and N-acetylcysteine. It exhibits therapeutic potential in PCS by targeting multiple pathway disorders including mitochondrial dysfunction, oxidative stress, and chronic inflammation (Finnigan et al., 2023). A phase IIa double-blind RCT demonstrated that 28 consecutive days of intervention significantly alleviated physical and mental fatigue in patients. The underlying mechanisms include promoting mitochondrial bioenergetic production, scavenging reactive oxygen species, suppressing pro-inflammatory factors such as IL-6 and TNF-α, improving endothelial function, and regulating fatty acid oxidation, thereby effectively relieving core symptoms such as persistent fatigue, exercise intolerance, and brain fog (Russell et al., 2024). Notably, objective assessment of mitochondrial function in calf muscle using advanced magnetic resonance spectroscopy revealed no overall differences between the treatment and placebo groups. This inconsistency between subjective symptomatic improvement and objective mitochondrial indicators suggests that the alleviation of fatigue may not fully rely on the objective recovery of mitochondrial function. Modulation of the ammonia-glutamine metabolic pathway may serve as an independent mechanism underlying the therapeutic effect of AXA1125.

As a dietary supplement, oxaloacetate is a key intermediate of the tricarboxylic acid cycle. It exerts therapeutic effects on PCS by enhancing mitochondrial biogenesis, restoring the balance of the NAD+/NADH ratio, and mitigating neuroinflammation (Fink et al., 2018). Existing studies have shown that oral oxaloacetate administration for 6 weeks markedly reduced physical and mental fatigue scores. In patients with long COVID, the fatigue improvement rate reached 46.8% in the 500 mg twice-daily group and 27.9% in the 1000 mg twice-daily group, showing a dose-dependent response. Its core mechanism involves reversing characteristic functional and metabolic disorders in post-COVID fatigue syndrome, including inhibiting aerobic glycolysis, restoring NAD+/NADH homeostasis, reducing NF-κB-mediated chronic inflammation, promoting mitochondrial biogenesis and energy metabolism, and activating the AMPK pathway to enhance glucose utilization. It also reduces ROS-induced damage through antioxidant effects, thereby ameliorating PCS via the integrated regulation of energy metabolism, oxidative stress, and inflammatory networks (Cash and Kaufman, 2022).

Nicotinamide adenine dinucleotide (NAD**+)** acts as an essential coenzyme for cellular energy metabolism and mitochondrial function. SARS-CoV-2 infection substantially consumes endogenous NAD**+** by activating the Poly ADP-ribose polymerase pathway, resulting in a marked reduction in NAD**+** levels and consequently exacerbating fatigue and cognitive impairment. Accordingly, NAD**+** supplementation represents a rational strategy for PCS intervention. Nicotinamide riboside serves as a key precursor of NAD**+**. A 24-week double-blind, placebo-controlled RCT enrolled 58 patients with long COVID. The results demonstrated that daily administration of 2000 mg nicotinamide riboside significantly elevated peripheral blood NAD**+** levels by 2.6–3.1-fold. Nevertheless, the main interventional effect on Fatigue Severity Scale (FSS) scores did not reach statistical significance. In an exploratory analysis integrating all participants after 10 weeks of intervention, FSS scores decreased by 3.77 points relative to baseline, suggesting that long-term continuous supplementation may exert a potential alleviating effect on symptoms (Wu et al., 2025). However, such exploratory findings cannot be regarded as definitive evidence of therapeutic efficacy. Nicotinamide riboside exhibits an overall favorable safety profile, and larger confirmatory trials are still required to verify its definite clinical efficacy against PCS.

Persistent symptoms of PCS are closely linked to mitochondrial function. The PINK1/Parkin-dependent mitophagy process is markedly suppressed under PCS conditions. Corresponding activators can break the vicious cycle from mitochondrial dysfunction to excessive inflammation by facilitating the clearance of damaged mitochondria (Luo et al., 2022). Urolithin A (UA), a natural mitophagy activator, is a metabolite produced by human gut microbiota following the degradation of pomegranates, certain berries, and nuts. Its capacity to enhance muscle function and combat fatigue has been widely validated in preclinical and clinical studies. UA significantly elevates mitochondrial activity and improves muscle function by activating PINK1/Parkin-mediated mitophagy. Accumulated evidence indicates that UA prolongs the lifespan of *Caenorhabditis elegans* and enhances muscle performance and endurance in humans and mice (Ryu et al., 2016). UA intervention effectively relieves the blockade of the PINK1/Parkin mitophagy pathway induced by long COVID-related inflammation and metabolic disorders. By clearing dysfunctional mitochondria and restoring mitochondrial homeostasis, UA exerts anti-fatigue effects, offering a novel therapeutic option for targeting mitochondrial impairment in PCS. Table 2 summarizes other candidate agents with mitochondrial protective potential.

**TABLE 2:** Other candidate drugs with mitochondrial protection potential.

Drug	Mechanism	References
Metformin	Activates AMPK and inhibits mitochondrial complex I to reduce mitochondrial ROS production	Cojocaru et al. (2023)
Curcumin	Activates Keap1/NRF2/ARE pathway, promotes NRF2 nuclear translocation, and upregulates activities of SOD, CAT and other antioxidant enzymes; inhibits NF-κB pathway to reduce release of TNF-α, IL-6 and other pro-inflammatory factors	Sahin et al. (2016), Cui et al. (2025)
Resveratrol	Activates AMPK/SIRT1 axis, deacetylates and activates PGC-1α, promotes mitochondrial biogenesis, and upregulates NRF2	Chen J et al. (2025)
Quercetin	Promotes NRF2 nuclear translocation, upregulates NRF2-related mRNA expression, and increases activities of SOD, GSH-Px, and CAT; inhibits mitochondrial apoptosis through the NRF2/HO-1 axis	Rondanelli et al. (2023), Zhang et al. (2024)
MitoQ	Protects mitochondrial DNA and proteins from oxidative damage and maintains mitochondrial respiratory chain efficiency	Lu et al. (2008)
Creatine	Accelerates ATP synthesis from ADP; activates AMPK signaling to upregulate PGC-1α expression and preserve mitochondrial structural and functional integrity	Barbieri et al. (2016)
Acetyl-L-carnitine	Transports long-chain fatty acids into the mitochondrial matrix to promote β-oxidation and ATP production; directly scavenges ROS, enhances SOD/GPx activity, and protects mitochondrial membranes against oxidative damage. Improves energy metabolism by restoring mitochondrial fatty acid β-oxidation and oxidative phosphorylation	Helbing et al. (2024)
Rapamycin	Upregulates the PINK1/Parkin pathway, facilitates clearance of damaged mitochondria, and reduces the release of ROS and mtDNA.	Ruan et al. (2025)

#### 4.1.4 Potential drugs targeting RAS imbalance

RAS imbalance represents one of the key mechanisms driving PCS. Dysregulation of the dual RAS axis establishes the pathological basis for sustained PCS progression through chronic inflammation, skeletal muscle lesions, and mitochondrial energy dysfunction (Holmes et al., 2024). Accordingly, targeting this pathway is regarded as a promising intervention strategy. Table 3 summarizes potential therapeutic agents and their underlying mechanisms that target the RAS system to ameliorate PCS.

**TABLE 3:** Potential therapeutic drugs and mechanisms of targeting RAS system to improve PCS.

Treatment strategy	Drug	Mechanism	References
Inhibit ACE/Ang II/AT1R axis	losartan, valsartan, lisinopril	Restores RAS system balance, reduces systemic inflammation levels; improves microcirculation and tissue perfusion, and alleviates fatigue induced by chronic inflammation and hypoxia	Cojocaru et al. (2023)
Activate ACE2/Ang 1–7/Mas axis	Vitamin D3	Relieves chronic low-grade inflammation, improves mitochondrial function and emotional regulation, and directly alleviates fatigue. Low serum vitamin D levels are correlated with PCS severity	Charoenporn et al. (2024)
	ω-3 fatty acids	Downregulates pro-inflammatory cytokines including IL-6 and TNF-α, mitigates systemic low-grade inflammation, facilitates the elimination of chronic inflammation via inflammatory mediators, and thereby alleviates inflammation-related fatigue	Arnardottir et al. (2021)
	Recombinant human ACE2	Restores the activity of the RAS protective pathway, exerts vasodilatory, anti-inflammatory, and anti-fibrotic effects, and potentially improves tissue energy metabolism and alleviates fatigue	Khan et al. (2017)
Inhibit ADAM17	ADAM17 inhibitor	Inhibits key pro-inflammatory factors such as TNF-α and IL-6, improves systemic low-grade inflammation, and relieves fatigue	Liu L et al. (2025)

Although strategies targeting RAS imbalance for PCS treatment are theoretically appealing, the evidence supporting their clinical translation remains insufficient. Therefore, despite RAS system dysregulation being a critical candidate mechanism underlying PCS, current evidence does not support the routine use of RAS modulators such as ACEI/ARB for PCS management. Rigorous RCTs with PCS as the primary endpoint are urgently needed in future research. Screening specific biomarkers for patients driven by aberrant RAS pathway activity will help clarify the true therapeutic value of targeting this axis in PCS.

#### 4.1.5 Regulatory strategies for MGB axis dysfunction

Patients with PCS exhibit persistent gut microbiota dysbiosis, predominantly characterized by depletion of beneficial SCFA-producing bacteria and enrichment of pro-inflammatory microbial taxa. The consequent reduction in SCFA levels acts as a core metabolic link by which MGB axis disruption drives chronic inflammation and neurocognitive symptoms (Lau et al., 2025). Homeostatic imbalance of the MGB axis constitutes an important pathological basis of PCS. Dysregulated MGB axis function is accompanied by reduced SCFA production, impaired enteroendocrine cell activity, and increased intestinal permeability. These alterations facilitate the entry of pathogens or lipopolysaccharide into the circulation, trigger the release of pro-inflammatory cytokines, and further exacerbate fatigue manifestations (Iqbal et al., 2025). Therefore, modulating gut microbial homeostasis through probiotic supplementation and dietary interventions may serve as an effective strategy for PCS management.

Probiotics exert antiviral effects by enhancing natural killer cell activity, promoting the generation of regulatory T cells and antimicrobial peptides, and boosting antibody production. Relevant studies have shown that supplementation with probiotics such as *Lactobacillus* and *Bifidobacterium* alleviated COVID-19 symptom severity by 51%, with significant improvements in fatigue, cognitive impairment, and headache (Neris Almeida Viana et al., 2024). In addition, probiotic and prebiotic formulations containing *Bifidobacterium* strains can regulate gut microbiota composition in patients 5 weeks after COVID-19 infection. Such interventions reduce viral load and decrease circulating levels of inflammatory mediators including IL-6, monocyte chemoattractant protein-1, macrophage colony-stimulating factor, IL-1 receptor antagonist, and TNF-α (Liu et al., 2022b), indicating that restored SCFA production can mitigate systemic inflammatory responses. Another study confirmed that the abundance of butyrate-producing bacteria, including *Faecalibacterium*, *Clostridium butyricum*, *Clostridium leptum*, and *Lactobacillus reuteri*, is markedly diminished in COVID-19 patients (Tang et al., 2020). As a major SCFA, butyrate exerts anti-fatigue effects via anti-inflammatory action, modulation of energy metabolism, and regulation of gut-brain axis signaling (Guo et al., 2023). Therefore, restoring intestinal microecological balance via supplementation with butyrate-producing strains or direct butyrate administration can alleviate chronic inflammation and MGB axis dysfunction, thereby relieving PCS symptoms. Other potential therapeutic agents targeting PCS through the MGB axis are summarized in Table 4.

**TABLE 4:** Potential therapeutic agents for ameliorating PCS via the MGB axis.

Drug	Mechanism	References
SIM01	Regulates gut microbiota composition, elevates the abundance of SCFA-producing bacteria, facilitates SCFA synthesis, and reduces antibiotic resistance genes	Rathi et al. (2021)
ImmunoSEB and ProbioSEB CSC3	As a systematic enzyme and probiotic complex, it alleviates PCS via multiple mechanisms including gut-brain axis regulation, anti-inflammation, antioxidation and immunomodulation	Parate and Shah (2021), Rathi et al. (2021)
VSL#3®	A multi-strain probiotic preparation containing eight strains (*B. breve, B. longum, B. infantis, L. acidophilus, L. plantarum, L. casei, L. bulgaricus, S. thermophilus*) alleviates PCS by restoring intestinal microecological homeostasis, enhancing intestinal barrier function, producing anti-inflammatory SCFA, and modulating the gut-brain axis	Marinoni et al. (2023)
Paraprobiotics	Acts as a non-viable preparation, modulates immune cell activation and TLR2 expression on T cells	Docampo et al. (2024)
Combined supplementation of vitamin K2 and vitamin D3	Vitamin K2 activates matrix Gla protein, while vitamin D3 exerts immunomodulatory and anti-inflammatory effects, thereby improving intestinal barrier function and inflammatory biomarkers	Atieh et al. (2025)
Glutamine	Serves as the primary metabolic fuel for intestinal epithelial cells, upregulates tight junction proteins to repair intestinal barrier, and reduces microbial translocation and systemic inflammation	Coqueiro et al. (2019), Durante (2023)
IDO1/TDO2 inhibitors	Inhibits excessive activation of the KP pathway and reduces neurotoxic metabolites such as 3-HK and quinolinic acid	Badawy (2023)
L-tryptophan	Replenishes tryptophan to restore serotonin and melatonin synthesis, counteracting KP-mediated neurotoxicity	Al-Hakeim et al. (2023)

Furthermore, dietary modulation may help improve overall health status and ameliorate gut dysbiosis in PCS patients. The Mediterranean diet is well recognized for its anti-inflammatory properties and is rich in dietary fiber, complex carbohydrates, omega-3 fatty acids, vitamins, and phytochemicals (Tsigalou et al., 2020). A study involving 612 individuals adhering to the Mediterranean diet reported significant reductions in pro-inflammatory markers such as C-reactive protein and interleukin-17 (Cassidy, 2020). The Mediterranean diet promotes the enrichment of SCFA-producing taxa including *Faecalibacterium prausnitzii* and *Eubacterium*, whose depletion has been well documented in patients with long COVID (Liu et al., 2022b).

Although the therapeutic potential of restoring SCFA homeostasis has been preliminarily demonstrated, large-scale RCTs with SCFA metabolites as predefined endpoints and standardized microbiota detection protocols are still required to clarify its definite clinical role in PCS treatment.

### 4.2 Potential drugs of other mechanisms

#### 4.2.1 Fluvoxamine

Fluvoxamine is a selective serotonin reuptake inhibitor. Owing to its anti-inflammatory effects primarily mediated via the sigma-1 receptor, it has been proposed as a therapeutic agent against COVID-19. Existing evidence indicates that oral administration of fluvoxamine at a daily dose of 100 mg for 10 days during the acute phase of mild-to-moderate COVID-19 can modulate post-infection immune dysregulation and neuroinflammation through multiple mechanisms, including activation of the sigma-1 receptor, suppression of inflammatory cytokine release, reduction of microvascular thrombosis formation, and retardation of melatonin degradation. This intervention markedly lowers the risk of core fatigue symptoms associated with post-COVID syndrome at the 12-week follow-up (Farahani et al., 2023), providing preliminary evidence for early pharmacological intervention to reduce the incidence of PCS. A recent large-scale, multicenter, randomized, placebo-controlled adaptive trial further validated the therapeutic efficacy of fluvoxamine for PCS (Reis et al., 2026). This trial enrolled 399 long COVID patients with fatigue lasting ≥90 days, who received either 100 mg fluvoxamine twice daily or placebo for 60 days. Compared with placebo, fluvoxamine significantly reduced FSS scores at day 60 and day 90, accompanied by sustained improvements in quality of life. The fluvoxamine group showed a lower incidence of adverse events, with rare serious adverse reactions. This finding provides high-level evidence supporting fluvoxamine for PCS treatment, yet the 90-day follow-up period limits evaluation of its long-term efficacy.

#### 4.2.2 Coenzyme Q10 combined with alpha-lipoic acid

Coenzyme Q10 (CoQ10) and alpha-lipoic acid (ALA) are two types of mitochondrial nutrients with synergistic effects. They play critical roles in repairing mitochondrial damage by improving electron transport chain function, scavenging ROS, and promoting mitochondrial biogenesis. Their application in long COVID and related syndromes has attracted increasing attention in recent years. A prospective observational study conducted by Barletta et al. first reported the therapeutic efficacy of CoQ10 combined with ALA in patients with post-COVID syndrome. The study enrolled 174 individuals with long COVID, among whom 116 patients in the treatment group received combined therapy with 100 mg CoQ10 and 100 mg ALA for 2 months. The results showed that 53.5% of patients in the treatment group achieved a complete response in the FSS with a reduction of no less than 50%, compared with only 3.5% in the control group. The non-response rate of FSS was merely 9.5% in the treatment group, which was significantly lower than 25.9% in the control group (Barletta et al., 2023). Nevertheless, this study adopted an observational design rather than a randomized double-blind controlled trial, which is insufficient to establish a definite causal relationship. Another RCT with a phase II randomized crossover design compared the efficacy of high-dose single-agent CoQ10 with placebo in PCS patients (Hansen et al., 2023). This RCT provides an important supplement to upgrade the evidence level for these two agents in PCS intervention.

#### 4.2.3 Cacao flavonoids

A recent randomized, double-blind, placebo-controlled trial evaluated the intervention efficacy of epicatechin-rich cacao flavonoid supplements in patients with PCS. After 90 days of intervention, the supplement significantly improved plasma levels of inflammatory markers (IL-1β, IL-6, TNF-α) and endothelial dysfunction marker syndecan-1, alongside remarkable improvements in fatigue scale scores and grip strength compared with the placebo group (Munguía et al., 2026). Although the exact mechanisms remain to be fully elucidated, the anti-inflammatory and antioxidant properties of flavonoids may contribute to fatigue relief, providing a novel dietary intervention strategy for PCS management. Notably, despite the encouraging preliminary results, the relatively small sample size (n = 46) warrants further verification in large-scale studies. Future research should also explore the optimal dosage and treatment course of flavonoids, as well as their potential synergistic effects with other therapeutic regimens.

#### 4.2.4 Amantadine

Amantadine is a centrally acting agent with dopaminergic effects and NMDA receptor antagonistic properties. Given its well-established efficacy in alleviating fatigue in patients with multiple sclerosis, amantadine has been proposed as a potential therapeutic option for PCS. Harandi et al. conducted an open-label randomized trial enrolling 83 patients with long COVID. The intervention group received amantadine treatment for 2 weeks, while the control group received no intervention. Fatigue severity was assessed via the Visual Analog Fatigue Scale and FSS. No significant between-group differences in baseline fatigue scores were observed. After 2 weeks of intervention, patients in the amantadine group exhibited substantial reductions in both scale scores, which were significantly superior to those in the control group (*P* < 0.001). The drug was well tolerated with no notable safety concerns (Harandi et al., 2024). Although preliminary findings indicate that amantadine effectively ameliorates PCS, the open-label and non-placebo-controlled design necessitates further validation via large-scale, double-blind, placebo-controlled trials.

#### 4.2.5 L-arginine combined with vitamin C

L-arginine, a semi-essential amino acid and nitric oxide precursor, is recognized as a promising non-antiviral therapeutic candidate in a comprehensive review on alternative therapies for long COVID (Livieratos et al., 2024). Its anti-inflammatory and immunomodulatory properties have garnered increasing attention. Accumulating evidence suggests that L-arginine supplementation, especially when combined with antioxidants such as hydroxytyrosol, can mitigate inflammation, oxidative stress, and immune dysregulation (Pérez de la Lastra et al., 2023). A single-blind, randomized, placebo-controlled trial evaluated the efficacy of L-arginine combined with vitamin C in 46 adult long COVID patients with persistent fatigue. At day 28, only 8.7% of participants in the active group reported fatigue, compared with 80.1% in the placebo group (*p* < 0.0001) (Tosato et al., 2022). These findings suggest that L-arginine combined with vitamin C supplementation serves as a safe and effective nutritional strategy to relieve persistent fatigue in adult long COVID patients. However, the single-blind design and limited sample size (n = 46) restrict the generalizability of the results. The short intervention duration and absence of long-term follow-up leave the durability of its efficacy unclear. Furthermore, the liposomal vitamin C adopted in this trial cannot be directly equated with conventional vitamin C preparations, and the independent contribution of L-arginine cannot be distinguished from the synergistic effect of vitamin C. Large-scale, double-blind, placebo-controlled trials with long-term follow-up are required to validate these preliminary outcomes and clarify the underlying mechanisms.

#### 4.2.6 Intravenous immunoglobulin

Intravenous immunoglobulin (IVIg) is an immunomodulatory therapy prepared from pooled human plasma and has been widely applied in autoimmune and inflammatory diseases. Current evidence for IVIg in PCS treatment is primarily derived from case reports and small-scale clinical studies. A case report documented a PCS patient who achieved significant clinical improvements in fatigue, cognitive impairment, myalgia, and dyspnea following IVIg treatment, accompanied by favorable alterations in immunological parameters (Camici et al., 2026). The authors proposed that IVIg exerts beneficial effects by neutralizing pathogenic autoantibodies, modulating T cell function, and regulating inflammatory responses. A prospective study involving 21 long COVID patients demonstrated marked alleviation of fatigue symptoms after three courses of monthly IVIg infusion (0.5 g/kg) (Seibert et al., 2024). Additionally, high-dose IVIg represents a promising therapeutic approach for ameliorating pulmonary, neurological, and cardiac symptoms of long COVID. Patients receiving high-dose IVIg reported significant improvements, including reduced fatigue, increased energy levels, and relieved chest pain (Khalid et al., 2026). A case series of 9 long COVID patients also confirmed the prominent clinical benefits of long-term high-dose IVIg therapy on fatigue and brain fog (Thompson et al., 2023).

Despite these encouraging results, current evidence supporting IVIg for PCS remains limited to case reports and small uncontrolled studies, precluding definitive conclusions. Its high cost, intravenous administration route, and potential adverse effects also restrict widespread clinical application. Large-scale controlled studies are urgently needed to establish the efficacy, safety, and optimal patient selection criteria for IVIg in PCS treatment.

#### 4.2.7 Antihistamines

Antihistamines may alleviate PCS by blocking histamine H1 and H2 receptors and inhibiting mast cell activation (Baptista et al., 2026). A single non-RCT investigated the efficacy of combined fexofenadine and famotidine in PCS treatment, including 14 patients in the treatment group and 13 in the control group. After 20 days of treatment, 29% of PCS patients achieved complete resolution of symptoms, and all treated patients exhibited significant improvements in core symptoms such as fatigue and brain fog relative to the control group (Salvucci et al., 2023). Antihistamines generally possess a well-established safety profile when used as directed. Nevertheless, no systematic reviews or rigorous RCTs have comprehensively validated the efficacy of antihistamines for PCS. The ongoing STIMULATE-ICP trial, which contains nested drug trials testing famotidine and loratadine, is expected to provide more robust clinical evidence upon completion.

#### 4.2.8 Astragalus root extract

Astragalus root extract exerts definite intervention effects on PCS. A triple-blind RCT demonstrated that nurses suffering from chronic fatigue after SARS-CoV-2 infection received 500 mg astragalus root extract twice daily for 30 consecutive days. The prevalence of chronic fatigue decreased from 100% to 13.8% at the end of intervention, and remained at 17.2% 1 month after the intervention. These values were significantly lower than those observed in the placebo group, where the prevalence was 80.0% at the end of treatment and 70.0% 1 month later, with all statistical differences reaching *P* < 0.0001 and revealing sustained therapeutic efficacy. Astragalus root extract can effectively ameliorate PCS with no obvious adverse reactions and satisfactory safety, suggesting that it can be used as a potent herbal preparation for alleviating PCS (Banihashemi et al., 2025). Regrettably, the specific mechanism by which astragalus root extract relieves PCS has not been further investigated in relevant research. According to the collation of existing literature, astragalus root extract can regulate cytokine IL-2 expression and facilitate the polarization of γδT cells toward a Th1-type immune response, thereby presenting a positive immunomodulatory trend. Meanwhile, astragalus extract possesses strong free radical scavenging capacity and can effectively relieve symptoms related to chronic fatigue syndrome (Latour et al., 2021). Existing studies have confirmed that excessive accumulation of free radicals acts as an important trigger for physical fatigue. The flavonoid and saponin active ingredients contained in astragalus not only underlie its efficacy against chronic fatigue syndrome but also serve as the major bioactive components responsible for free radical scavenging *in vivo* (Lee and Song, 2015).

In addition, a real-world observational study among long COVID patients has verified the fatigue-relieving effect of Myelophil, which is formulated as a 1:1 compound preparation of Astragalus and Salvia miltiorrhiza extracted with 30% ethanol. The study enrolled 50 participants including 18 males and 32 females, and 49 of them were included in the intention-to-treat analysis. Significant statistical improvement in fatigue status was observed after 4 weeks of continuous administration (Joung et al., 2024). Furthermore, a phase 2 clinical trial further supported the clinical potential of Myelophil (Joung et al., 2019), confirming its prominent benefits for treating ME/CFS, especially in patients with severe symptoms. A subsequent observational study evaluated MYPplus, an enhanced formulation containing agarwood added to the original Astragalus and Salvia miltiorrhiza formula, in 50 patients with long COVID. Four weeks of treatment yielded significant improvements in fatigue and quality of life, indicating that the addition of agarwood may further boost the clinical benefits of the basic Myelophil formula in alleviating PCS and cognitive impairments (Choi et al., 2025). Furthermore, various medicinal plants and compound formulations exhibit potential for improving PCS, and their mechanisms of action are summarized in Table 5.

**TABLE 5:** Potential medicinal plants, compound formulas and their mechanisms against PCS.

Medicinal plants	Mechanism	References
Ginseng	Regulates carbohydrate metabolism, activates PI3K/Akt/mTOR signaling pathways, enhances mitochondrial energy supply efficiency in skeletal muscle and accelerates free radical scavenging, thereby exerting anti-fatigue effects	Lu et al. (2021), Zhou et al. (2022), Zheng et al. (2023)
HRG80 Red Ginseng	Restores impaired mitochondrial function: reduces lactic acid and lactate dehydrogenase levels, improves mitochondrial density and morphology in skeletal muscle, elevates activities of Na^+^-K^+^-ATPase and cytochrome c oxidase, and activates the AMPK/PGC-1α pathway	Zhang H et al. (2022)
Rhodiola rosea	Elevates levels of superoxide dismutase and nerve growth factor, reduces C-reactive protein, enhances oxygen uptake and carbon dioxide excretion, thereby alleviating chronic fatigue syndrome	Ishaque et al. (2012), Song et al. (2015), Zhang et al. (2021), Ivanova Stojcheva and Quintela (2022)
Ginkgo biloba	Exerts multi-target pharmacological effects including antioxidant, anti-inflammatory, anti-apoptotic and vasoregulatory activities, and alleviates PCS by mitigating neuroinflammation, oxidative stress and mitochondrial dysfunction	Mueller and Müller (2024)
Maca	Reduces levels of harmful metabolites such as blood urea nitrogen and lactic acid, and relieves fatigue by elevating the NAD^+^/NADH ratio	Zhu et al. (2021)
Bu-Zhong-Yi-Qi decoction	It targets various inflammation-related proteins including IL-1β, IL-6, TNF-α, COX-2, iNOS, CHRM1, OPRM1, MAPK3 and VEGFA. Among them, the inhibitory effects on IL-6, IL-1β and TNF-α are particularly crucial for PCS. It also enhances SOD activity and reduces MDA and ROS levels	Li et al. (2022), Zhang J et al. (2022)
Dang-Gui-Shao-Yao San	Reduces the levels of multiple inflammatory mediators including IL-2, IL-1β, TGF-β, RAGE and TLR2; scavenges free radicals, inhibits lipid peroxidation and elevates SOD activity	Chen Y et al. (2025)
Ling-Gui-Zhu-Gan decoction	It possesses antioxidant properties, reduces the production of MDA and ROS, enhances SOD activity, and regulates the balance between oxidants and antioxidants	Wang et al. (2020)
Qingjin Yiqi granules	Enhances SOD activity, decreases MDA and ROS levels, and upregulates mRNA expression of Akt and NRF2 in skeletal muscle	Pang et al. (2022)

## 5 Conclusion and discussion

Compared with previously published reviews on PCS, the present review has three distinct innovations. First, most prior studies have separately described immune dysregulation, mitochondrial dysfunction, RAS imbalance, and gut microbiota dysbiosis as independent pathological mechanisms. In contrast, this review highlights that these four core mechanisms interact synergistically through sustained excessive inflammation and collectively form a self-sustaining vicious cycle, rather than functioning in isolation. This integrated framework provides a more integrated explanation for the persistent nature of PCS even after viral clearance. Second, while most existing reviews merely elaborate pathological mechanisms, the present review establishes a comprehensive “mechanism–target–drug” research paradigm, offering clearer scientific insights for future PCS drug development and clinical translation. Third, unlike previous reviews that generally focus on a single category of therapeutic agents, this study comprehensively categorizes candidate interventions according to their underlying mechanisms, covering chemical drugs, nutritional supplements, probiotics, and herbal medicines, thereby constructing a more holistic therapeutic framework for PCS.

Based on this integrated framework, this review summarizes the four core pathological mechanisms underlying PCS, which are sustained by chronic inflammation following viral clearance. These mechanisms include immune dysregulation and chronic inflammation, mitochondrial dysfunction and impaired energy metabolism, RAS imbalance, as well as gut microbiota dysbiosis and MGB axis disorder. Based on these mechanisms, this review rigorously screened relevant targets, including those associated with the TLR4/TLR7, JAK/STAT pathway, NLRP3 inflammasome, AMPK/NRF2 axis, PINK1/Parkin pathway, RAS, and MGB axis. On this basis, the current status of clinical evidence for each candidate drug was evaluated. At present, candidate agents such as fluvoxamine, LDN, coenzyme Q10 combined with α-lipoic acid, AXA1125, and SIM01 synbiotics have shown preliminary improvement signals to varying degrees.

Although the aforementioned studies provide a preliminary direction for the intervention of PCS, several limitations in current research warrant attention. First, most existing studies are still based on a cross-sectional design, and the interactions between various pathological mechanisms need to be further elucidated through longitudinal studies. Second, current intervention strategies mainly rely on single-target drugs, which are difficult to fully address the networked and mutually reinforcing nature of PCS pathological mechanisms. Third, compared with the progress in mechanism research, the clinical advancement of drug interventions is relatively lagging. Moreover, current evidence is mainly derived from observational studies or small-sample RCTs, which is insufficient to be regarded as definitive evidence of efficacy, and additional confirmatory studies are still required. Fourth, the selection of outcome indicators in current experimental studies has not been standardized; different scales are used to assess fatigue, cognitive function, and quality of life, which hinders the integration of evidence across trials.

In view of the above problems, future research can be advanced in the following directions. First, promote longitudinal multi-omics studies by integrating plasma proteomics, metabolomics, fecal metagenomics, PBMC transcriptomics, and other multi-omics technologies to comprehensively elaborate on the pathological mechanisms of PCS. Second, explore multi-target combination intervention strategies, such as combinations of AMPK activators and mitophagy inducers, JAK inhibitors and NAD+ precursors, as well as immunomodulators and probiotics/synbiotics, to enhance therapeutic efficacy. However, the interactions and potential risks between different drugs still need to be evaluated in preclinical models. Third, more large-scale, multicenter studies focused on PCS should be conducted in the future to consolidate the efficacy evidence of existing candidate drugs. Fourth, consensus on the core indicators of PCS should be promoted, and biomarkers should be introduced on this basis. Candidate biomarkers include the activation level of the NLRP3/IL-1β/IL-18 axis, kynurenine/tryptophan ratio, serum SCFA concentration, and Ang II/Ang 1-7 ratio, enabling candidate drugs to accurately match patient subgroups most likely to benefit.

Beyond the identification of therapeutic targets and screening of candidate agents, translating mechanistic findings into clinical practice remains a major challenge. A recently proposed practical primary care framework for long COVID management provides a structured solution to this issue (Brode and Melamed, 2024). This four-step framework encompasses energy pacing strategies to prevent post-exertional malaise, intentional rehabilitation targeting physical, cognitive, and emotional impairments, symptom management via pharmacological and non-pharmacological interventions, and individualized cautious administration of experimental targeted therapies. The framework emphasizes personalized treatment based on patients’ dominant symptom profiles and encourages adaptive updates in accordance with evolving scientific evidence. Notably, the fourth step involving experimental therapeutic trials aligns well with the mechanism-targeted candidate agents summarized in this review. This indicates that the translation from mechanistic targets to clinical application relies not only on drug development, but also on feasible clinical delivery systems. Future studies should prioritize integrating emerging pharmacological evidence into such clinical frameworks to facilitate the timely and safe implementation of novel therapies in primary care settings.

In conclusion, the research on the pathogenesis of PCS has established a relatively comprehensive pathological framework centered on the maintenance of chronic inflammation after viral clearance. The screening of therapeutic targets and the clinical validation of candidate drugs are progressing in an orderly manner. Currently, the field is in a critical stage of transition from mechanism exploration to clinical verification. The core issues urgently to be addressed are the insufficient depth of mechanism verification and the lack of clinical evidence. In future research, multi-target combination intervention regimens may become a promising therapeutic direction for improving PCS.
